# Widespread Lateral Transmission in *Fergusonina* Galling Flies (Diptera: Fergusoninidae) and Their Obligate Nematode Mutualists Does Not Preclude an Overall Pattern of Cospeciation

**DOI:** 10.1002/ece3.73511

**Published:** 2026-05-28

**Authors:** Sonja J. Scheffer, Matthew L. Lewis, Kerrie A. Davies, Robin M. Giblin‐Davis, Gary S. Taylor, Leigh A. Nelson, Matthew F. Purcell, Jeff R. Makinson, Weimin Ye, Kevin E. Omland, David K. Yeates

**Affiliations:** ^1^ USDA‐Agricultural Research Service Systematic Entomology Lab Beltsville Maryland USA; ^2^ School of Agriculture, Food and Wine, Centre for Evolutionary Biology and Biodiversity University of Adelaide Adelaide South Australia Australia; ^3^ Fort Lauderdale Research and Education Center University of Florida/IFAS Fort Lauderdale Florida USA; ^4^ Centre for Evolutionary Biology and Biodiversity, and School of Biological Sciences University of Adelaide Adelaide South Australia Australia; ^5^ Grains Research and Development Corporation (GRDC) Kingston Australian Capital Territory Australia; ^6^ USDA‐ARS Australian Biological Control Laboratory Indooroopilly Queensland Australia; ^7^ North Carolina Department of Agriculture and Consumer Services Raleigh North Carolina USA; ^8^ Department of Biology University of Maryland Baltimore County Baltimore Maryland USA; ^9^ CSIRO National Research Collections Australia Canberra Australian Capital Territory Australia

**Keywords:** cophylogeny, endosymbionts, galling insects, horizontal transmission, mutualism, vertical transmission

## Abstract

Cospeciation between symbionts or other tightly associated organisms is believed to occur primarily in the case of strict vertical transmission of the interaction from parents to offspring. In a unique and obligate mutualism, *Fergusonina* flies and *Fergusobia* nematodes together form galls on plants in the Myrtaceae, primarily in Australia. The intimate biology of this interaction strongly suggests the presence of strict vertical transmission of nematodes from female flies to daughters. We obtained both fly and nematode mitochondrial sequences from extractions from 136 female flies from 118 galls of *Fergusonina daviesae*, *Fergusonina omlandi*, and *Fergusonina taylori*, all of which feed on overlapping hosts and are broadly sympatric to syntopic. In each of these three focal species, there were many cases of multiple fly haplotypes associated with a single nematode haplotype and vice versa. In the haplotype networks and phylogenies within each species pair, the only case of related fly haplotypes being exclusively associated with related nematode haplotypes was for *F. taylori* where there was a largely concordant split between fly and nematode haplotypes from Tasmania and mainland Australia. Despite strong evidence of widespread lateral transfer of nematodes within fly species, there was no direct evidence of heterospecific transfer of nematodes. Consistent with this, phylogenetic analyses of 29 *Fergusonina* and *Fergusobia* pairs found highly concordant fly and nematode phylogenies indicative of an evolutionary history dominated by cospeciation. In *Fergusonina* and *Fergusobia,* what appears to be widespread breakdown of strict vertical transmission within several species does not preclude substantial cospeciation in these groups.

## Graphical abstract

The biology of gall‐forming fergusoninid flies and their obligate nematode mutualists suggests strict vertical transmission of nematodes between fly generations. Using mitochondrial sequence data to associate fly and nematode haplotypes, we found widespread intraspecific horizontal transfer in multiple species. Nevertheless, a strong pattern of cospeciation in the broader clades containing these and other species was found, indicating a decoupling of intraspecific and interspecific processes.

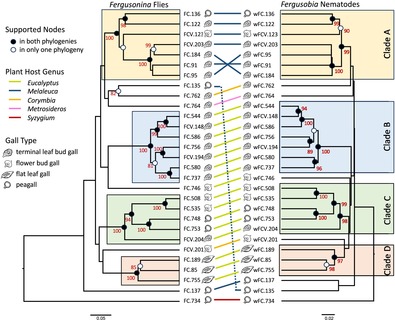

Coevolution in the form of cospeciation can be a powerful explanation for diversification in closely associated organisms (Page 2003). Cospeciation does not require reciprocal evolutionary change as in one‐to‐one coevolutionary change, but rather repeated instances of contemporaneous divergence (Winkler and Mitter 2008). Multiple lines of evidence are required to document a history of cospeciation, including topological and temporal concordance in the phylogenies between clades of ecologically associated organisms. Although cospeciation has been looked for in many groups, most examples have been found in closely associated symbioses and parasitisms (Peek et al. 1998; Funk et al. 2000; Hosokawa et al. 2006; Winkler and Mitter 2008).

It is well‐established that the key characteristic of cospeciating organisms is strict vertical transmission of the interaction (Page 2003; Hosokawa et al. 2006; Cruaud and Rasplus 2016). Parents pass the interaction directly to offspring in each generation, which then pass the interaction to their offspring. Vertical transmission of an interaction typically allows little to no opportunity for offspring to become associated with other intra‐ or interspecific lineages. In contrast, lateral (or horizontal) transmission occurs when the interaction is not conserved across generations. Instead, descendants break the relationship by becoming associated with other lineages. If horizontal transfer occurs across species, the evolutionary trajectories of the interacting species can become uncoupled. Such interspecific horizontal transfer can result in host shifts among symbiont participants, as well as lead to other discordant events (Paterson and Gray 1997; Page 2003; Breusing et al. 2019).

Life histories and the details of the biology and ecology of interactions between interacting organisms are drivers of the specificity of transmissions and the likelihood and prevalence of cospeciation (Clayton and Johnson 2003; Clayton et al. 2003; Weckstein 2004; Hosokawa et al. 2006). The precise details of parent‐offspring transmission including patterns of dispersal are critical. Organisms in obligate associations involving close physical contact, for example a variety of organisms and their associated bacteria, having strict vertical transmission are most likely to exhibit patterns of cospeciation (Funk et al. 2000; Hosokawa et al. 2006; Groussin et al. 2020). Most metazoan organisms that have seemed likely candidates for strict cospeciation have proven not to be, particularly upon reanalysis and new methods (de Vienne et al. 2013). This includes some well‐known systems including mutualistic fig‐fig wasps and parasitic feather lice and their avian hosts. Given their generally close relations with their host plants, herbivorous insects have seemed especially good candidates for diversifying with their host plants. However, few studies of plant‐feeding insects have uncovered instances of strict cospeciation with their plant hosts (Winkler and Mitter 2008; de Vienne et al. 2013; Suchan and Alvarez 2015). To a large extent, the host divergences are dramatically older than the divergences in associated insect lineages (Winkler and Mitter 2008). In addition, even recently diverged plants and insects are unlikely to cospeciate because, in almost all species, strict vertical transmission of host plant‐insect association does not occur. In most species, insects have an at least minimally vagile juvenile and/or adult life stage capable of choosing different host individuals and even species from those used by their parents.

Here we investigate the relationship between the occurrence of vertical transmission currently occurring within species of an obligate fly‐nematode mutualism and the degree of cospeciation among the interacting taxa over evolutionary time.

## 1 Study System

**FIGURE 1:**
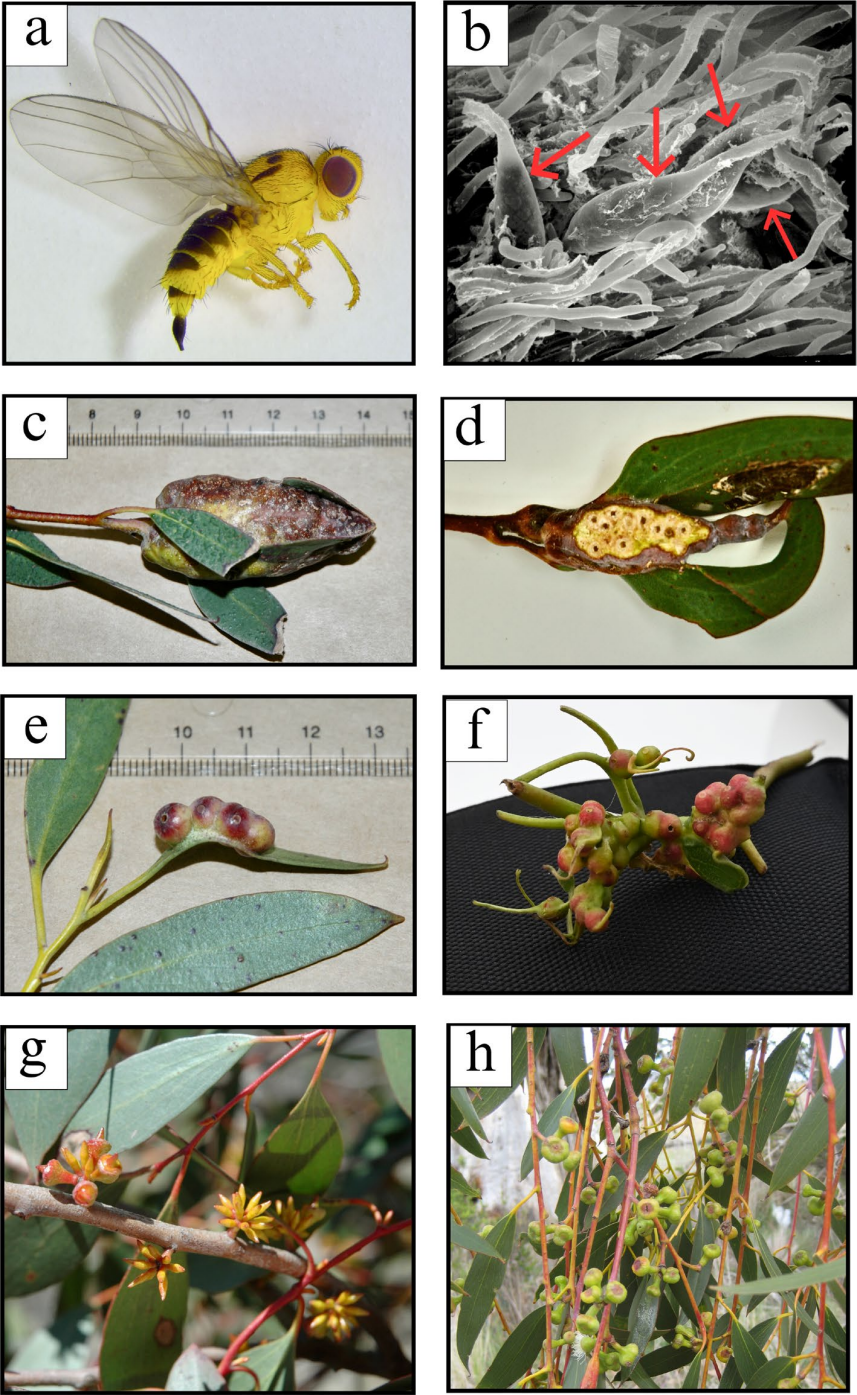
*Fergusonina*, *Fergusobia*, and diversity of galls on snow gum and other host plants: (a) female *Fergusonina taylori*, (b) SEM of four fly eggs (indicated by red arrows) and associated nematodes within a host bud, (c) *Fergusonina* sp. M‐Shoot Bud gall on snow gum, (d) dissected locules of a M‐Shoot Bud gall of *Fergusonina omlandi* on snow gum, (e) M‐Leaf Peagalls on snow gum, (f) M‐Peagalls on unknown *Eucalyptus*, (g) M‐Flower Bud gall clusters on *Eucalyptus stellulata* : Galled flower buds to the far left, ungalled buds are slender, (h) numerous *Fergusonina* sp. M‐Flower Bud galls on snow gum.

*Fergusonina* flies (Diptera: Fergusoninidae) and *Fergusobia* nematodes (Nematoda, Neotylenchidae) together form galls on plants in the myrtle family (Myrtaceae) in a unique and obligate mutualistic association (Figure 1) (Currie 1937; Davies et al. 2016). *Fergusonina* and *Fergusobia* have been found primarily in Australia and all feed on plant species in the Myrtaceae. Seven genera are known to host *Fergusonina/Fergusobia* including *Eucalyptus, Melaleuca, Angophora*, *Corymbia, Syzygium*, and *Leptospermum*, and one species on *Metrosideros* in New Zealand. One species is known from India on *Corymbia* (Sidiqi 1986). Approximately 42 *Fergusonina* fly and 50 *Fergusobia* nematode species have been described, with nearly 100 additional associations known (Nelson et al. 2014; Scheffer et al. 2017; the authors, unpub. data). This is undoubtedly a tremendous underestimate of diversity in these groups as only a fraction of plants in the host genera have been sampled for *Fergusonina*‐*Fergusobia* galls. This is particularly true for *Eucalyptus* of which there are more than 700 species in Australia. Many host plant species harbor multiple different galling pairs, and some widespread plant species, such as *E. camaldulensis* and *E. pauciflora s.l*. (see below), have been found to host as many as eight *Fergusonina‐Fergusobia* species pairs, often in sympatry, making multiple types of galls (Figure 1) (Nelson et al. 2014; Scheffer et al. 2017).

The *Fergusonina*‐*Fergusobia* galling mutualism is an obligate symbiosis in which each partner derives benefit. It involves the initiation by *Fergusobia* nematodes of the formation of galls on host plant tissue, within which nematodes and fly larvae both feed independently (Giblin‐Davis et al. 2001; Giblin‐Davis, Center, et al. 2004). Upon completion of development within the gall, all female flies emerge carrying nematodes within their abdomens to the next host site where fly eggs and nematode juveniles are deposited. Thus, in this partnership, the nematode provides establishment of the protected structure (gall) for nematode and larval fly feeding, and the fly provides transport to new host plant feeding sites (Giblin‐Davis et al. 2001; Davies et al. 2010). No female flies or developing fergusoninid galls have been found without *Fergusobia* nematodes, and no *Fergusobia* nematodes have been found making galls without *Fergusonina* (Giblin‐Davis et al. 2001). No other insects and no other nematodes are known to form an obligate plant‐parasitic mutualism together.

That the *Fergusonina*‐*Fergusobia* interaction is an old and stable one as indicated by (1) the large molecular distances across extant species of both groups (Ye et al. 2007; Scheffer et al. 2017, this study), (2) morphological and molecular isolation from other fly and nematode groups (McAlpine et al. 1981; Giblin‐Davis, Scheffer, et al. 2004; Ye et al. 2007; Bayless et al. 2021), and (3) simply the large number of species pairs in the context of (1) and (2), above. The biology of these groups is unusual, and both groups are evolutionarily isolated within the larger taxa to which they belong.

The unique life history of the *Fergusonina*‐*Fergusobia* nematode symbiotic mutualism has been detailed elsewhere (e.g., Giblin‐Davis et al. 2001; Giblin‐Davis, Davies, et al. 2004; Giblin‐Davis, Scheffer, et al. 2004; Davies et al. 2010, 2016; Nelson et al. 2014). To summarize, all female *Fergusonina* flies carry *Fergusobia* nematodes within their abdomens. These include obese reproductive adult female nematodes in a parasitic form, their eggs, and hatched juvenile *Fergusobia* nematodes. Upon oviposition, female flies deposit their own eggs along with juvenile nematodes (Figure 1). The nematodes deposited by female flies into oviposition sites initiate gall formation, during which period 1 to > 200 (depending on species) locule chambers lined with specialized hypertrophied cells are formed (Giblin‐Davis, Davies, et al. 2004). Each locule contains one fly egg or larva along with a complement of free‐living *Fergusobia* nematodes. Within each locule the single fly larva and the nematodes feed separately, the nematodes have a parthenogenic generation, and then produce a sexual generation followed by mating (Nelson et al. 2014). It is not until the fly larva reaches the 3rd and final instar prior to pupation that mated pre‐parasitic female nematodes enter the larvae destined to be female flies (Currie 1937; Fisher and Nickle 1968; Scheffer et al. 2013). *Fergusobia* nematodes do not enter (or persist) in larvae/pupae destined to be male, nor are they transported on adult male flies in some other way. This has been confirmed by molecular screening for nematodes of various fly life stages of the flies: all female flies and one‐half of the large larvae/pupae were positive for nematodes, while all male flies and one‐half of the large larvae/pupae were negative (Scheffer et al. 2013), consistent with decades of observation (Currie 1937; Fisher and Nickle 1968; Giblin‐Davis, Scheffer, et al. 2004).

Upon emergence from the galls, all female flies carry mated female nematodes in their parasitic form. These absorb fly haemocoel and generate large numbers of eggs, which hatch to become the juvenile nematodes that will be deposited during fly oviposition (Giblin‐Davis et al. 2001). It is these juvenile nematodes that initiate gall formation around the eggs of the flies, thus completing the life cycle of this mutualism. The obligate and lineage‐specific transmission of *Fergusobia* nematodes between fly generations suggests that this interaction is one of strict vertical transmission of the nematodes across generations, from female flies to daughters.

Galls of different species of *Fergusonina‐Fergusobia* differ considerably in morphology and in the location and host tissue used (Figure 1) (Currie 1937; Giblin‐Davis, Center, et al. 2004; Davies et al. 2010; Nelson et al. 2014). Galls of different types also vary in the number of individual fly larvae that occur within each. For example, many species form unilocular single galls having only one occupant, some flower galls and flat leaf galls have a dozen or fewer, whereas multilocular terminal bud galls can have dozens to hundreds of occupants (Nelson et al. 2014). Unilocular galls are almost certainly founded by single females, but large multilocular galls can be formed by multiple female foundresses (Purcell et al. 2015). In the case of galls being founded by multiple females, it is not known whether the nematodes become intermixed and may become associated with eggs other than those of their host fly or whether the eggs and nematodes from each female fly remain associated within individual locules. If, instead, the nematodes do become intermixed, the possibility of lateral transmission of nematodes arises as nematodes become affiliated with fly eggs from novel females. If there is heterospecific founding of galls, lateral transmission of nematodes between fly species could result in a host‐shift of nematodes, a nonconcordant evolutionary event.

Separate phylogenetic analyses of *Fergusonina* flies and *Fergusobia* nematodes have uncovered some degree of general phylogenetic concordance (Ye et al. 2007; Scheffer et al. 2017). This is unlikely to be due to cospeciation between the host plants and either the flies or the nematodes as the estimated ages of the host plant genera within the Myrtaceae are much older than those estimated for the origin of the Fergusoninidae and are not especially related (Nelson et al. 2014; Scheffer et al. 2017; see Thornhill et al. 2019). Even considering only “tip” clades of fly and host plant phylogenies, there is little evidence of fly‐host plant cospeciation (Purcell et al. 2018).

The available fly and nematode phylogenies show primarily specialized species interspersed with fewer more oligophagous species (Ye et al. 2007; Scheffer et al. 2017; Purcell et al. 2018). There is some correspondence in host plant affiliations as evidenced by the presence of a large *Melaleuca* terminal leaf/shoot bud gall clade as well as other, smaller, host plant‐associated clades in both the fly and nematode trees. Both the fly and nematode phylogenies indicate repeated host shifts between distant plant groups as well as numerous recolonizations of original plant lineages (Ye et al. 2007; Scheffer et al. 2017). While these similarities in host plant‐use patterns of the flies and the nematodes suggest the possibility of fly‐nematode cospeciation, the phylogenies in these two studies cannot be rigorously compared as the species included are not all the same. In addition, for those species in common between the studies, the individual flies and nematodes may have been from different collections such that the specimens were not true associates. This could result in misleading patterns of incongruence in the fly and nematode phylogenies. The opportunity for mis‐association is substantial in this system as multiple unrelated *Fergusonina‐Fergusobia* species can form the same type of galls on the same hosts even in sympatry (Figure 1) (Davies et al. 2010; Nelson et al. 2011a; Scheffer et al. 2017).

To investigate cospeciation in *Fergusonina*‐*Fergusobia* mutualists, we chose a multitiered approach, looking first at intraspecific patterns of nematode transmission within three related fly species found in various degrees of sympatry. We asked whether strict vertical transmission of nematodes occurs within these species by determining the associations between fly and nematode haplotypes obtained from DNA extractions from single female flies. One‐to‐one fly‐nematode haplotype associations would be consistent with strict vertical transmission. A stronger test is whether related fly haplotypes are associated with related nematode haplotypes such that association by descent is likely. Second, we asked whether heterospecific haplotype associations were present that might indicate past or current lateral transmission of nematodes between fly species. We then broadened our approach to assess the overall pattern of cospeciation in these groups by comparing the phylogenetic congruence between the focal *Fergusonina* and *Fergusobia* species pairs. These occur in broad sympatry, and two fly species, *F. daviesae* Nelson and Yeates and *F. taylori* Nelson and Yeates, and associated nematodes form large galls on the high‐altitude snow gums at the same sites, while the third fly, *F. omlandi* Nelson and Yeates, occurs on the same or similar host plant at lower elevations in the same region (Nelson et al. 2011a, 2011b). Finally, we assess the overall pattern of cospeciation within *Fergusonina* and *Fergusobia* by comparing the topological congruence of 29‐species fly and nematode phylogenies.

## 2 Materials and Methods

For the broad phylogenetic work, *Fergusonina*‐*Fergusobia* galls were collected over a decade from *Eucalyptus, Melaleuca, Corymbia, Metrosideros*, and *Syzygium* species throughout Australia (including one species from New Zealand). For the haplotype‐association work involving *F. daviesae, F. taylori*, and *F. omlandi*, targeted collecting from “snow gums” ( *E. pauciflora* and close relatives) took place in eastern Australia (Table 1). The terminology of Booker and Kleinig (1999) is used here, and distributions of the eucalypt species are listed in Atlas of Living Australia (ALA 2023).

**TABLE 1:** Geographic and altitudinal variation in collecting sites for galls used in this study. Distributions, collection location, dates, elevation, and species occurrences of sympatry from specimens of *F. tasmaniensis* , *F. omlandi*, *F. daviesae* , and *F. taylori* reared from snow gums *E. pauciflora s.l.* and *E. coccifera* .

Location	State	Coll. Date	Elev. (m)	Ferg. tasmani.	Ferg. omlandi	Ferg. daviesae	Ferg. taylori	Sympatry
Brindabellas		Jan. 2008	?		2			
Edmonst. Rest Area	NSW	Sept. 2007	753		2			
Captain's Flat	NSW	Sept. 2007	767		10			
Lyell Hwy	TAS	Oct. 2007	772					
Avoca	TAS	Oct. 2007	Low	4				
Rt 526	TAS	Oct. 2007	Low	3				
Great Lake	TAS	Oct. 2007	1022				9	
Mt Field ( *E. coccifera* )	TAS	Oct. 2007	1045				2	
Mt Wellington ( *E. coccifera* )	TAS	Oct. 2007	1152				4	
20 km neDinner Plains	VIC	Sept. 2007	1292		1			
Mt Buffalo	VIC	Sept. 2007	1472				2	Y
Pt. Lookout	NSW	Sept. 2007	1546		9			
Dead horse Gap	NSW	Sept. 2007	1573			6	1	Y
Dinner Plains	VIC	Sept. 2007	1588			3	9	Y
Pryor's Hut	ACT	Nov. 2007	1650			5	24	Y
Mt. Ginini	ACT	Nov. 2007	1675			4		
Falls Creek Village	VIC	Sept. 2007	1675			14		
Mt Gingera	ACT	Nov. 2007	1700			44	3	Y
Mt Hotham	VIC	Sept. 2007	1742			10	5	Y
Perisher	NSW	Sept. 2007	1911			3		

*Eucalyptus pauciflora* is a widespread species with at least seven recognized subspecies. The most widespread is *E. p. pauciflora* which occurs throughout its range at mid and low altitudes. The remaining subspecies have highly restricted geographic ranges and can only be recognized by slight differences in reproductive tissues that may or may not be present, and all have common names referencing “snow gum”. Several subspecies are restricted to high elevations of the southeastern mountains in the Australian states of the Australian Capital Territory (ACT), New South Wales (NSW), and Victoria (VIC). Here we use *E. pauciflora* s.l. to refer to all the pauciflora subspecies including the highly restricted range species *E. gregsoniana* and *E. lacrimans*, both once considered subspecies of *E. pauciflora* . However, the Tasmanian snow gum, *E. coccifera* , is distinctive. Likely host species and subspecies of snow gum collections are presented in Table 1. None of the nematodes associated with the flies collected from the terminal bud galls on snow gums have yet been described.

Galls were collected and placed in self‐sealing plastic bags. It is not currently possible to distinguish which *Fergusonina* fly species made particular terminal bud galls on snow gum without inspection of the adults or DNA barcoding. For this reason, all galls were taken and our gall sample size for each fly species represents relative abundance at a particular site. As flies emerged from the galls, they were placed into 95% ethanol and stored in a −80°C freezer. Only female flies were used for DNA extractions. For haplotype associations, both fly and nematode sequence data were collected from each DNA extraction, ensuring that unambiguous haplotype associations came from each fly and the nematodes it carried. For the majority of galls, only a single female was used in this study to ensure that our samples represent population‐wide variation without potential effects of non‐independence due to gall resampling. However, multiple females were analyzed from a subset of galls to investigate whether galls involving these species have multiple female foundresses.

Total nucleic acids were extracted using the Qiagen DNeasy Blood & Tissue kit (Qiagen, Valencia, CA). From each genomic sample, a region of mitochondrial cytochrome oxidase (COI from both flies and nematodes) was amplified separately using fly and worm taxon‐specific primers (Appendix Table A1). COI PCR product was enzymatically purified using ExoSAP‐IT (Affymetrix, Santa Clara, CA, USA). PCRs for the nuclear genes carbamoyl phosphate synthetase (CAD) and 6‐phosphogluconate dehydrogenase (PGD) co‐amplify fly and nematode amplicons using fly primer combinations CAD54F‐CAD405R and PGD2F‐2.5R. The double bands representing smaller fly amplicons and larger nematode amplicons were gel purified using QIAquick Gel Extraction kits (Qiagen, Valencia, CA) for sequencing. Sequencing reactions were carried out using Big Dye Terminator v3.1 Sequencing kits (Applied Biosystems, Foster City, CA) using the amplifying primers (Appendix Table A1). Sequencing reactions were cleaned by ethanol‐precipitation and analyzed on an ABI 3130XL or 3730XL Genetic Analyzer. Sequences were assembled and edited with Sequencher (Gene Codes Corp., Ann Arbor, MI) and aligned using Geneious Prime (2024: www.geneious.com).

All DNA sequences have been deposited into GenBank. Specimen collection information, fly and nematode haplotype designations, and GenBank accession numbers for the haplotype association study are in Appendix Table A2. Collection and GenBank information for specimens in the phylogenetic study are in Appendix Table A3.

### 2.1 Haplotype Associations

For each of the three fly focal species, *F. daviesae* , *F. omlandi*, and *F. taylori* , the mitochondrial COI sequences from individual flies and their associated nematodes were composed into two single representation haplotype alignments. For each of the six resulting haplotype alignments, median joining haplotype networks were constructed in PopArt using the default epsilon value of 0 (Bandelt et al. 1999; Leigh and Bryant 2015). To visualize haplotype associations with the context of haplotype networks, the nematode haplotypes were coded by color and mapped onto the associated fly networks. In addition, maximum likelihood (ML) trees were generated for fly and nematode datasets for each species using IQ‐tree (Nguyen et al. 2015) with 1000 UltraFast bootstrap replicates. These phylogenies are presented as tanglegrams (Page 2003).

To investigate the pattern of interspecific haplotype association within the context of cospeciation across the snow gum (+*coccifera*) species, all fly and associated nematode COI data were pooled by taxon into two multispecies datasets. For these analyses, we included fly and nematode sequences from five *F. tasmaniensis* specimens, the fourth terminal bud galling species on snow gums.

### 2.2 Phylogenetic Concordance in *Fergusonina* and *Fergusobia*

To investigate phylogenetic concordance in the evolutionary history of *Fergusonina* and *Fergusobia*, we constructed phylogenies from 29 fly‐nematode pairs spanning the known species diversity (see Ye et al. 2007; Scheffer et al. 2017). For each group, sequence data from mitochondrial, CAD, and PGD genes (fly: COI/COII 1329 bp, CAD 659 bp, PGD 701 bp; nematode: COI 480 bp, CAD 258 bp, PGD 947 bp) were concatenated into a single dataset. These were analyzed with IQ‐Tree using default parameters, and 1000 UltraFast bootstrap values were generated. ModelFinder (Kalyaanamoorthy et al. 2017) was used to identify the optimal substitution model for the flt analysis as GTR + F + I + G4 and for the nematode analysis as TIM3 + F + I + G4. In fly and nematode analyses, the *Sygyzium* feeders were used as the outgroup. This was chosen because in a previous analysis of *Fergusonina* including outgroups from other fly families, this species was sister to the remaining *Fergusonina* species. Appropriate outgroups for *Fergusobia* are not clear, and in any case, we had no access to reasonably appropriate candidates. For this reason, the *Fergusobia* tree was rooted with the associated *Szyzgium*‐feeding nematode. Because this article is focused on topological congruence, choice of outgroup will not affect the conclusions as long as the same species‐pair is used in both instances.

The event‐based analysis software Jane (Conow et al. 2010) with default parameters was used to estimate the number of cospeciation, host shift, duplication, and loss events necessary to reconcile the nematode phylogeny with that of the fly. Because portions of the results of the Jane analysis are biologically implausible, we also present modified graphical representations of more reasonable scenarios.

## 3 Results

**FIGURE 2:**
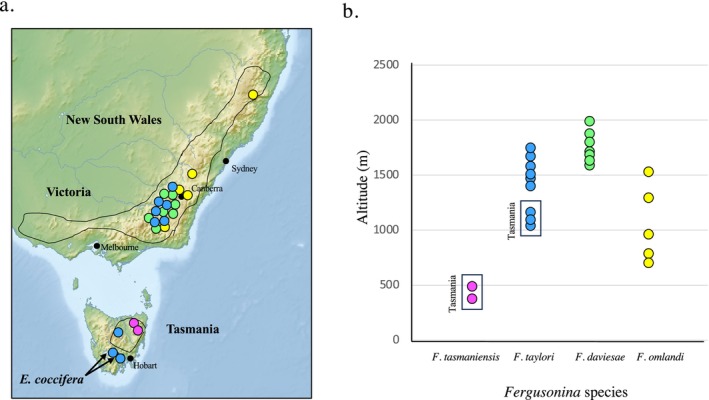
Geographic and altitudinal distributions of snow gums and sampling of *Fergusonina‐Fergusobia* species in this study: (a) geographic distributions; (b) altitude (m). The two sampling locations involving *E. coccifera* are indicated by black arrows. Map from Google Maps.

Galls were collected from *Eucalyptus pauciflora s.l*. and *E. coccifera* at altitudes ranging from 211 m in Tasmania to 1911 m near the Perisher ski area in New South Wales (Figure 2 and Table 1). Fly and nematode mitochondrial COI sequences were obtained from female specimens from 129 galls. These included 88 *F. daviesae* , 23 *F. omlandi*, and 38 *F. taylori* galls (Table 2). An additional five individuals from two galls of *F. tasmaniensis* were used in the snow gum phylogenetic analysis. Multiple females were sampled from 22 galls for a total of 82 flies to assess whether more than one fly haplotype may be present within single galls (Table 3).

**TABLE 2:** Sample sizes and measures of diversity in the three focal fly species, *F. daviesae* , *F. omlandi*, and *F. taylori* and their associated nematodes. N Galls is the number of galls the samples are from and F/N Pairs is the number of matched flies and nematode pairs. These are followed by COI sequence length, the number of haplotypes, maximum pairwise distance, nucleotide and haplotype diversity.

Fly species	# Galls	F/N pairs	COI bp	# Haps	Max PWD	Nuc Div	Hap Div
*F. daviesae*	68	88					
Fly			649	33	2.16	0.00619	0.86834
Nematode			475	14	1.14	0.00122	0.41849
*F. omlandi*	23	24					
Fly			673	18	1.34	0.00525	0.95652
Nematode			496	8	1.00	0.00324	0.73188
*F. taylori*	38	38					
Fly			644	33	2.02	0.00904	0.95733
Nematode			386	8	5.18	0.01817	0.71823

**TABLE 3:** Galls from which nematodes from multiple flies were sequenced. Laboratory gall code, fly species, collection location, the number of flies sequenced, and the number of distinct nematode haplotypes within each gall are given. Max PWD presents the greatest pairwise difference in fly haplotypes found within each gall. The length of COI used for comparisons within galls is indicated.

Gall	Species	Location	*N* flies	*N* Nem Hap	Max PWD	bp
07–189	*F. daviesae*	Mt Hotham Village	3	1	0	688
07–190	*F. daviesae*	Mt Hotham Village	3	1	0	743
07–201	*F. daviesae*	Dinner Plains	2	1	0	733
07–284	*F. daviesae*	Dinner Plains	3	1	0	702
07–377	*F. daviesae*	Mt. Gingera	2	1	0	739
07–385	*F. daviesae*	Mt. Gingera	19	3	1.45	689
07–114	*F. omlandi*	Captain's Flat	2	1	0	730
07–116	*F. omlandi*	Captain's Flat	2	1	0	718
07–122	*F. omlandi*	Captain's Flat	2	1	0	738
07–158	*F. omlandi*	Captain's Flat	2	1	0	728
07–186	*F. omlandi*	20 km e Dinner Plains	2	1	0	738
07–265	*F. omlandi*	20 km e Dinner Plains	3	1	0	721
07–270	*F. taylori*	Dinner Plains	3	2	1.68	714
07–283	*F. taylori*	Dinner Plains	2	2	0.22	717
07–330	*F. taylori*	Tas, Great Lake	2	1	0	715
07–328	*F. taylori*	Tas, Great Lake	2	1	0	720
07–332	*F. taylori*	Tas, Great Lake	2	2	0.28	720
07–355	*F. taylori*	Pryor's Hut	16	2	0.78	643
07–356	*F. taylori*	Mt. Gingera	2	1	0	737
07–370	*F. taylori*	Pryor's Hut	3	1	0	752
07–290	*F. tasmaniensis*	Tas, Rd. C526	2	2	0.13	1537
07–300	*F. tasmaniensis*	Tas, w Avoca	3	2	0.07	1424

The fewest number of mitochondrial haplotypes within fly species was 18 for *F. omlandi*, and 33 for both *F. daviesae* and *F. taylori* (Table 2). Haplotype diversity was high in all three species (Table 2). Nucleotide diversity in the fly species was highest in *F. taylori* . Maximum intraspecific pairwise distances were 2.16% for *F. daviesae* , 1.34% for *F. omlandi*, and 2.02% for *F. taylori* (Table 2). Of the 22 galls from which multiple females were sequenced, seven contained more than one fly haplotype, with a maximum of three haplotypes. Pairwise COI distances of haplotypes from flies sampled from the same gall ranged from 0.12% to 1.68%. Nematode haplotypes were collected from 41 females from 5 galls, and all nematode haplotypes within each gall were identical.

The number of mitochondrial nematode haplotypes found in the three species ranged from 8 to 14 (Table 2). Haplotype diversity in nematodes from *F. daviesae* was roughly half of those from the other species. Variation in nucleotide diversity spanned an order of magnitude between nematodes associated with *F. daviesae* compared to those associated with *F. taylori* . Maximum pairwise distances within nematode species were 1.1% for *F. daviesae* associates, 1.0% for associates of *F. omlandi*, and 5.2% for *F. taylori* associates (Table 2).

### 3.1 Fly‐Nematode Haplotype Associations

**FIGURE 3:**
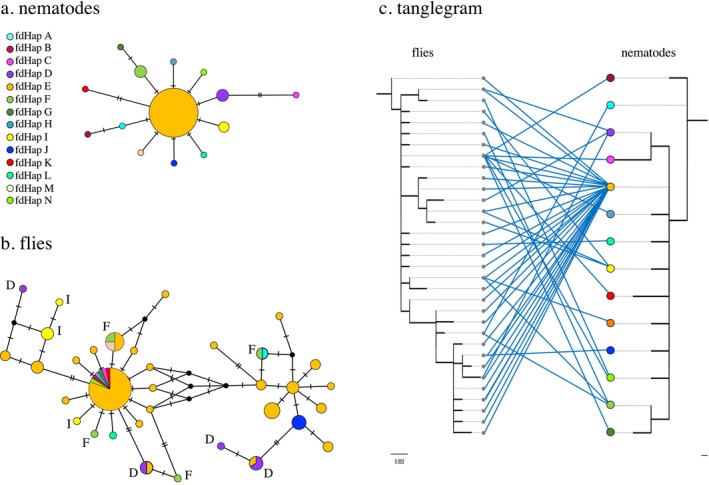
Genetic structure of *Fergusinina daviesae* and its associated *Fergusobia* sp.: (a) haplotype network of *Fergusobia*; (b) haplotype network of *F. daviesae* colored by nematode haplotype; (c) tanglegram of associations between fly and nematode haplotypes.

**FIGURE 4:**
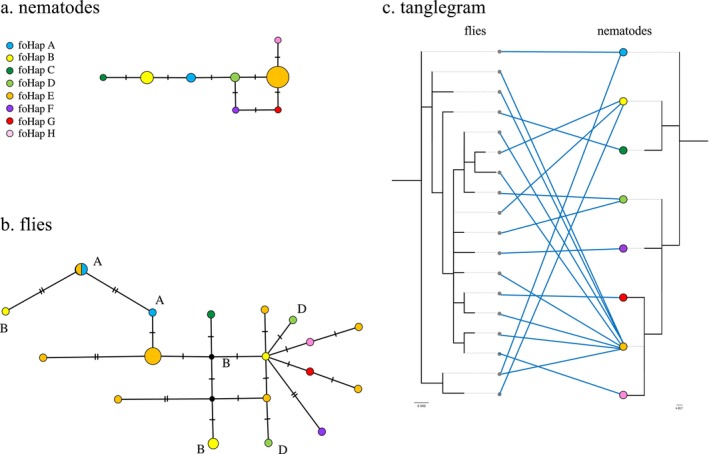
Genetic structure of *Fergusinina omlandi* and its associated *Fergusobia* sp.: (a) haplotype network of *Fergusobia*; (b) haplotype network of *F. omlandi* colored by nematode haplotype; (c) tanglegram of associations between fly and nematode haplotypes.

**FIGURE 5:**
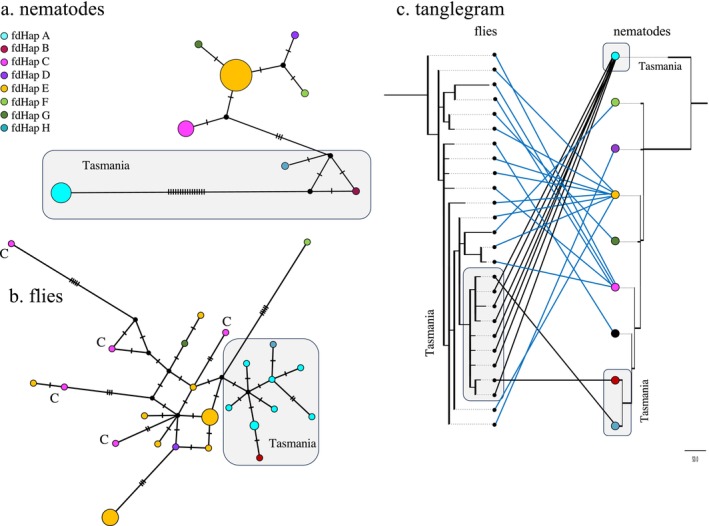
Genetic structure of *Fergusinina taylori* and its associated *Fergusobia* sp.: (a) haplotype network of *Fergusobia*; (b) haplotype network of *F. taylori* colored by nematode haplotype; (c) tanglegram of associations between fly and nematode haplotypes.

Comparison of fly and nematode COI haplotype sequences from 153 female flies found that in all three species fly and nematode haplotypes were correctly associated by species. Within species, fly and worm haplotypes did not show a strict one‐to‐one association (Figures 3, 4, 5). In all three nematode species, the most common nematode haplotype is shown in gold (Figures 3, 4, 5). Across all species, the ten nematode haplotypes represented by more than one individual were associated with more than one fly haplotype. In addition, for nine of these, the replicates were present in multiple unconnected locations in the fly haplotype networks, and do not form clusters (Figures 3, 4, 5). In the tanglegrams, a similar pattern can be seen of multiple nematode haplotypes being associated with a single fly haplotype as well as the reverse (Figures 3, 4, 5).

**FIGURE 6:**
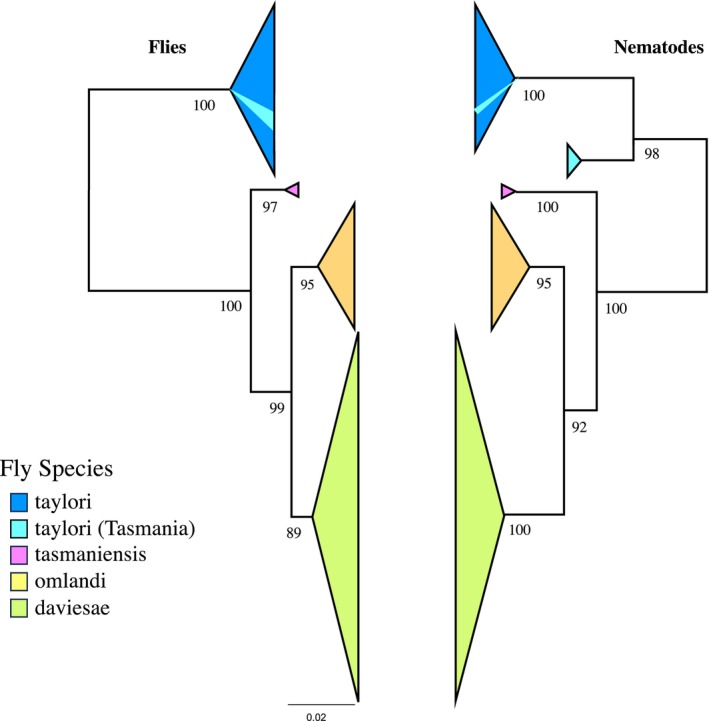
Maximum likelihood phylogenies for *Fergusonina* and *Fergusobia* M‐Shoot Bud gallers on snow gums. Bootstrap support values are shown below branches. Branch lengths of the two phylogenies are not to scale.

The networks and tanglegrams for each fly‐nematode species pair showed some degree of topological concordance. The *F. daviesae* flies and their nematode mutualists both exhibited star‐shaped networks (Figure 3). However, the fly network had additional structure that appeared to involve two groups separated by seemingly unsampled haplotypes. The *F. daviesae* haplotype networks differed substantially from the networks associated with *F. omlandi* (Figure 4) and *F. taylori* (Figure 5). Both haplotype networks from *F. omlandi* lacked a strong central haplotype and instead were primarily composed of singleton haplotypes linked by one or two steps. In *F. taylori* , the nine fly haplotypes from Tasmania formed a monophyletic group in both the haplotype network and in the tanglegram (Figure 6). In the nematode network, the Tasmanian haplotypes formed a cluster, but the most common Tasmanian haplotype (Haplotype A) was highly distinct from the remaining *F. taylori* (Figure 6). In the nematode phylogeny, Haplotype A was again distinct, but the two remaining Tasmanian haplotypes came out well within the phylogeny. There was no pattern of haplotype association with host plant species (Figure 6).

### 3.2 Cospeciation in *Fergusonina* and *Fergusobia*

**FIGURE 7:**
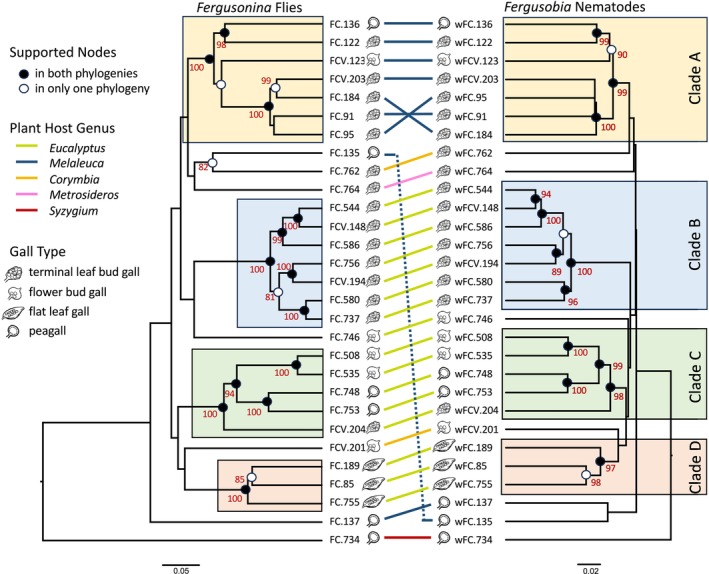
Maximum likelihood tanglegram comparing *Fergusonina* and *Fergusobia* phylogenies. Bootstrap support shown near supported nodes which are indicated by black dots. Nodes with moderate to high support in only one phylogeny are indicated by white dots. Well‐supported clades are within colored boxes.

Maximum likelihood phylogenetic analysis using COI, CAD, and PGD produced trees for *Fergusonina* and *Fergusobia* that were very similar. The trees had a number of well‐supported clades in common and low or no support for deeper nodes (Figure 7). The tanglegram involving the fly and nematode trees showed considerable parallelism (Figure 7). Twenty‐three of 29 species‐pairs were found within the same four well‐supported monophyletic (95%–100%) clades in both the fly and nematode trees (Figure 7). Of the 16 nodes within these four clades, 14 had bootstrap support above 90% in the fly phylogeny and 15 in the worm phylogeny.

**FIGURE 8:**
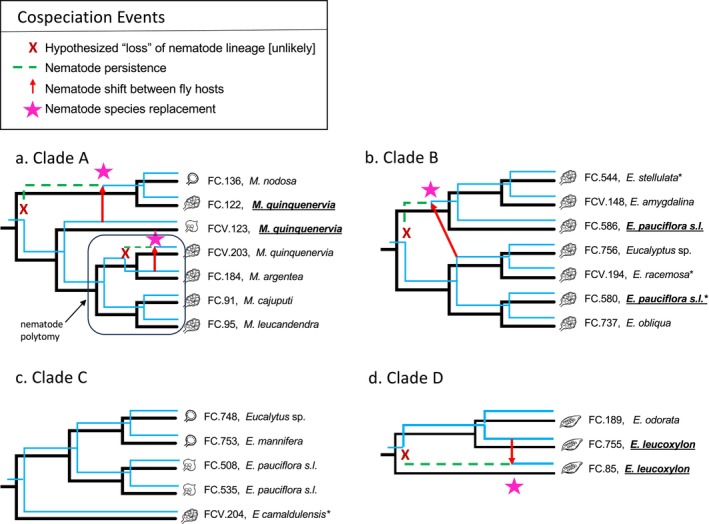
Reconciled phylogenies of well‐supported fly and nematode clades showing hypothesized shifting of nematodes between fly lineages.

Only four nodes within three of the clades were discordant between the fly and nematode trees (Figures 7 and 8). There were two cases of discordance in nodes among the seven species in Clade A. The first involved a resolved and supported node in the nematode tree uniting wFCV.123 as sister to wFC.136 + wFC.122, but an unsupported rearrangement involving species FCV.123 in the fly tree. A second discordance at the tip of Clade A involving FC.91 and FC.95 is not a real discordance but rather an apparent polytomy within the nematode node uniting these and related species. Clade B also consisted of seven species, and the fly and nematode trees differed in two nodes involving the placement of the pair wFC.756 + wFCV.194. The discordant nodes were poorly supported in both trees: 81% in the fly tree and 66% in the worm tree. Clade D contained only three species and a single discordant node. Clade C consisted of five species and the fly and nematode phylogenies were entirely concordant (Figures 7, 8). Note that we defined clades of interest as those with high levels of support. Bending this rule would allow Clades C + D to be merged for an increase in three concordant nodes in both phylogenies.

Cospeciation analysis of the three clades containing the discordant nodes using the software program Jane found that all can be categorized as a host shift event (Figure 8). Specifically, a transfer of nematodes from one fly lineage to a different fly lineage was postulated to reconcile the trees (Figure 8, red arrows). Note that in Clade B the transfer looks to be backwards in time, but this figure is a cladogram and has no implications for branch lengths or timing. Under Jane's scenario, the receiving branch of the fly tree is assumed to have no partner nematode lineage, having been lost at the red “X” (Figure 8). Because this is biologically unrealistic, we modified Figure 8 to indicate the more reasonable suggestion of continuity in the worm lineages (Figure 8, green dashed line), with replacement of the initial worm lineage with the new worm lineage at the time of the host shift (Figure 8, pink star).

As stated earlier, the tip clade of Clade A involves a nematode polytomy. This portion of Figure 8 is based on an essentially arbitrary resolution of this polytomy by the cospeciation analysis program Jane. Although this portion of Clade A is presented for completeness, we consider it spurious and do not address it further in this article.

To reconcile the fly and nematode trees in Clade A, it is postulated that nematodes from a lineage containing a flower bud galler on *M. quinquenervia* shifted to a fly lineage containing a terminal bud galler on *M. quinquenervia* and a pea galler on *M. nodosa* . In clade B, there was a shift of a nematode lineage between fly lineages having no host plants in common given the current sampling. In clade D, there was a shift between lineages ancestral to two flat leaf gallers on *E. leucoxylon* (Figure 8).

## 4 Discussion

Strict vertical transmission of closely associated organisms is viewed as a critical condition for tight cospeciation (Page 2003; de Vienne et al. 2013). Despite a life cycle that makes strict vertical transmission of *Fergusobia* nematodes with *Fergusonina* appear likely, our findings indicate that this is not necessarily the case, at least for galls containing multiple locules. In all three focal species there was not one‐to‐one correspondence of fly‐nematode haplotypes. Instead, individual nematode haplotypes were associated with multiple and unrelated fly haplotypes as shown in both the networks and the tanglegrams. The lack of correspondence of fly and nematode haplotypes is strong evidence of a breakdown in vertical transmission. That it occurs in multiple species indicates that this may be a common pattern, at least for large multilocular galls having multiple foundresses. Even if the most common nematode haplotype in each species (Haplotype E, gold color) is assumed to be ancestral, the overall lack of pattern still holds. The one exception is in *F. taylori* where the highly diverged nematode Haplotype A is shared by multiple related fly haplotypes from Tasmania. Given the ca 10,000 years of geographic disjunction between Tasmania and mainland Australia, it is perhaps not surprising to find Tasmania‐restricted groups along with genetic evidence consistent with incomplete lineage sorting. If populations across the Bass Strait are truly distinct with no migrants, it would be expected that the patterns of lineage sorting in both *F. taylori* and its nematodes will continue and eventually result in phylogenetically distinct monophyletic groups via allopatry.

Even without strict vertical transmission, the population dynamics of the nematodes are constrained within those of their fly symbionts. Nematode populations will necessarily experience the same overall population‐level forces as the flies. This includes events such as population bottlenecks or expansions, although their own levels of inbreeding and dispersal among fly lineages will influence the population genetic consequences. This raises the question: to what extent does population structure correspond between flies and associated nematodes in each species pair? We found that in *F. daviesae* there was overall congruence between the fly and nematode networks as both exhibited the distinctive star pattern of a common haplotype (two in the case of the flies) with single mutations to many rare haplotypes indicative of recent population expansion. In contrast, the correspondence between fly and nematode networks for both *F. taylori* and *F. omlandi* was less clear. In *F. omlandi*, the networks were primarily of rare haplotypes connected in series by single mutations, consistent with long periods of population stability. The networks in *F. taylori* also exhibited many connections of rare haplotypes in addition to the concordant groups of Tasmanian specimens.

In all cases of fly and nematode networks, haplotype variation within the nematode species was lower than its corresponding fly species. Although mutation rates of the flies and nematodes are unknown, the free‐living adult stage of the flies will allow dispersal and some large degree of panmixia. In contrast, the lifecycle of the nematodes causes them to experience a substantial degree of sib‐mating within locules, as well as repeated bottlenecks as large asexual within‐gall nematode populations are reduced to a much smaller number of pre‐infective sexual forms able to enter a pre‐female fly larva. In addition, nematodes may be under stronger stabilizing selection given the complexity of their lifestyles. Any of these processes can lower standing genetic variation.

The ecology and behavior of any particular interaction are important to processes of vertical and horizontal transmission (Johnson and Clayton 2004). Particularly important is whether there is a stage in an otherwise closely associated symbiosis where a breakdown in strict intraspecific vertical transmission can occur. In the *Fergusonina*/*Fergusobia* interactions studied here, a breakdown in strict intraspecific vertical transmission seems likely to be related to the type of galls made by these species (Nelson et al. 2011a). Multilocular terminal bud galls can be large and may include > 200 locules, each containing a fly larva and a group of nematodes. Galls of this type result from fly deposition of fly eggs and juvenile nematodes into the bud meristematic chamber by one or more females (Giblin‐Davis, Center, et al. 2004; Purcell et al. 2015; this study). With multiple foundresses, nematodes deposited by different females may be free to intermingle and become associated with fly eggs of other females as the gall locules form. However, if there is never or rarely any gall sharing by heterospecific *Fergsonina/Fergusobia* species, then horizontal transfer between offspring of conspecific female foundresses would have little to no effect on evolutionary processes of codivergence.

Various *Fergusonina* and *Fergusobia* pairs make other gall types on their host plants. These range from unilocular pea and stem galls having only a single larva and nematodes originating from a single female fly to various sizes of other multilocular galls on buds, flowers, leaves, and stems. The number of female foundresses that typically deposit their eggs and nematodes into a single oviposition site will determine the likelihood of strict vertical transmission in any particular species pair. We predict that future comparison of haplotype associations in species forming unilocular galls founded by single females will show a much greater degree of intraspecific vertical transmission and correspondence between fly and nematode haplotypes than that found here.

Although our results indicate substantial intraspecific horizontal transmission of *Fergusobia*, no nematode transfer between any of the focal fly species was found. This is despite the fact that we collected many *F. daviesae* and *F. taylori* in sympatry at the same sites at the same time. In addition, flies and nematodes of *F. omlandi* occurred in broad sympatry with these, present in the same mountains at lower elevations. Phylogenetic analyses of 153 paired samples of these three focal species of fly and associated nematode haplotypes indicated that all species of both fly and nematodes were monophyletic with no evidence of heterospecific transmission.

It seems remarkable that *F. daviesae* and *F. taylori*, which both oviposit into apical meristem shoot buds on the same host plants in the same locations and make the same gall types, would not occasionally share galls. Our direct data on gall sharing comes from 22 galls within which sampled *Fergusonina* flies were all conspecific. Purcell et al. (2015) found evidence of widespread conspecific female foundresses in the galls of four *Fergusonina* species. Despite frequent oviposition into a bud by conspecific females, our broader datasets show no evidence for a recent history of successful heterospecific nematode transfer that might arise from shared galls. Possibly there are species‐specific chemical deterrents preventing more than one species from ovipositing into the same gall location. In addition, there could be important chemical or morphological differences among host tree individuals that are important for fly oviposition such that fly species sharing a host plant species do not share host plant individuals. Myrtaceae are well known for their bioactive compounds (e.g., eucalyptus and tea‐tree oils). Many species possess distinct intraspecific variants (chemotypes) (Padovan et al. 2014; Saber et al. 2024) that might be variably attractive or deterrent to different insect species.

Although either mechanism seems possible for preventing heterospecific gall formation over ecological time frames, it is harder to envision their long‐term efficacy over evolutionary time. *Fergusonina*‐*Fergusobia* are a highly diverse set of interacting pairs with a history of extensive host plant shifting (Ye et al. 2007; Scheffer et al. 2017), necessarily implying some degree of oviposition lability in host use and occasional oviposition host expansions or “mistakes” (Larsson and Ekbom 1995). In addition, when considering Australia's variable ecological conditions, particularly fire and drought, oviposition sites involving meristematic tissues used by both species may be at least occasionally rare. That insects will oviposit on a substrate they would normally reject as has been shown in numerous oviposition experiments with other herbivorous insects, and this can vary depending on the degree of “motivation”, generally time since oviposition (Singer et al. 1992). On the face of it, it seems that oviposition into a rare meristematic tissue already occupied by eggs and larvae of a different species should be better than no oviposition at all.

A stronger hypothesis for the apparent lack of heterospecific gall sharing is that eggs and/or larvae and nematodes of different species may not be compatible. Our evidence for exclusively conspecific fly‐nematode associations is based entirely on reared *Fergusonina/Fergusobia* pairs. These are individuals that successfully formed a gall, fed, and completed a successful symbiotic life cycle. This includes mated nematodes entering the late‐stage fly larva, withstanding fly pupation, and discarding cuticle for absorption of fly abdominal hemocoel, all while ameliorating any active fly immune response. In using exclusively reared samples we only “see” compatible and successful fly‐nematode associations. This ignores any potential heterospecific dynamics and differential mortality that could occur within heterospecific shared galls. *Fergusonina daviesae* and *F. taylori* and associated nematodes on *E. pauciflora s.l*. are not particularly closely related, and it may be simplistic to assume that the nematodes are essentially interchangeable between the flies. Heterospecific associations may occur but not be viable, thus resulting in successful adult emergence of only flies having correct nematode associates.

### 4.1 Cospeciation

Phylogenetic analysis of 29 *Fergusonina* and *Fergusobia* species spanning the diversity of the fly family Fergusoninidae uncovered substantial fly‐nematode concordance. In the four clades that were well‐supported (bootstraps > 95%), only three convincing episodes of a host shift of nematodes between fly lineages were found. Two of these involved source and receiving lineages that were both in some way related to the current host plants of the fly‐nematode lineages. For example, within Clade A, there is a nematode host shift from a fly feeding on *M. quinquenervia* to a lineage of flies that includes a *M. quinquenervia* ‐feeding fly. Because we do not know the plant host affiliation(s) along the internal branches of the tree, it is not possible to definitively conclude that this shift of nematodes came about between fly species sharing the same host, *M. quinquenervia* . In the second case, in Clade D, there appears to be a direct shift of nematodes between two fly species currently feeding on *E. leucoxylon* . While seeming to represent a nematode shift between flies feeding on the same host plant species, a larger phylogeny of *Fergusonina* (Scheffer et al. 2017) shows that the clade containing these two species contains a number of additional species. The shift by nematodes may be deeper than appears here and may not be directly between fly species on *E. leucoxylon* . Nevertheless, a shift appears to have occurred directly between *E. leucoxylon* flies or at least to an ancestral fly lineage that may have been feeding on *E. leucoxylon* . With the current data it is not possible, in either case, to definitively identify the ancestral host plants involved with these transfers. However, the pattern observed in two of the three shifts suggests that host shifts by nematodes between fly species may be facilitated by the species identity of the host plant.

In all three cases of host shifts, the analysis program Jane inferred that an evolutionary loss of nematodes in the receiving fly lineage occurred prior to all three shifts. According to this model, nematodes in each of these three fly lineages were lost for some extended period. Subsequently, a lateral transfer of nematodes in the form of a host shift between lineages occurred, essentially a recolonization of the receiving fly lineage. Because the flies are dependent on the nematodes and are not found without them, this scenario is extremely unlikely to be accurate. A biologically realistic scenario is that there was no loss of nematodes until the host shift, at which point there was a replacement of nematodes rather than a recolonization of an “empty” fly lineage. The dynamics of such a replacement of one nematode species by another are not known, but some form of competition may occur, either within galls or among conspecific fly‐nematode pairs involving the new vs. the old nematodes. Alternatively, nematode identity may have a neutral effect on fitness of galling pairs, in which case the introduction of a new nematode into an established interaction would function as the equivalent of a new genetic mutation subject to genetic drift.

Patterns suggestive of cospeciation can arise from non‐coevolutionary processes (de Vienne et al. 2007; Suchan and Alvarez 2015). Host‐tracking, or pseudospeciation, occurs when host shifts and speciation by one lineage follows speciation of the other lineage based on chemical, morphological, or other characters. In such cases, the topologies of the two lineages may be concordant, but the timing of their diversifications will be different (Page 2003; de Vienne et al. 2007). In the case of *Fergusonina* and *Fergusobia*, the intimate and obligate biology of the association would seem to require a very convoluted host‐tracking evolutionary scenario to explain the strong pattern of cospeciation shown here. Neither flies nor nematodes are found to occur independently of each other. Samples of additional species coupled with genomic data and fully resolved trees would be ideal for more comprehensively addressing the question of how often and when during evolutionary time nematode transfer or host‐shift speciation occurs versus cospeciation. Of great interest will be whether clades of galls having multiple foundresses, such as terminal bud and flat leaf galls, have more instances of host switching events than do clades of unilocular galls that are the result of a single fly foundress.

Thousands of nematode species have relationships with insects, often parasitic, but numerous cases of phoresy and commensalism also occur (Giblin‐Davis et al. 2003; Sommer 2025). The best known are species of aphelenchoidid, diplogastrid, rhabditid and tylenchid nematodes associated with fig wasps, mushroom‐breeding flies, and some wood‐boring beetles (Massey 1974; van Goor et al. 2023; Kanzaki and Giblin‐Davis 2018). Currently, the tylenchid nematodes *Howardula* appear to be the closest relatives to *Fergusobia* (Ye et al. 2007). *Howardula* species associated with flies are in some ways quite similar to *Fergusobia*: they are internally phoretic, produce offspring within the adult fly abdomens, and are deposited into a new food source (mushroom) where they infest a new host larva. They differ ecologically from *Fergusonia* in that in *Howardula* transmission between generations is largely horizontal (within mushrooms), host‐fly specificity is not universal, and there is lability in host‐associations across diverse host taxa (Jaenike and Anderson 1992; Perlman et al. 2003). In addition, *Howardula* and its insect hosts do not represent a mutualism as there is no benefit known for the fly host, infection typically causes a reduction in female fertility, and infection rates fluctuate (Jaenike and Anderson 1992; Perlman et al. 2003). Given the similarities, it could be that the *Fergusonina‐Fergusobia* interactions evolved from nematodes having an initially phoretic relationship with flies (Giblin‐Davis, Scheffer, et al. 2004).

An alternative scenario is suggested by the fact that thousands of nematodes are parasitic on plants, and some form galls using complex combinations of morphological and molecular effectors (Siddiqi 2000; Giblin‐Davis et al. 2003; Baldwin et al. 2004; Gheysen and Mitchum 2011, 2019). Phytophagy and gall‐forming by nematodes raise a second possibility for the origin of the *Fergusonina‐Fergusobia* interaction. It is known that an undescribed fly in the related family Agromyzidae almost exclusively forms leafmines within galls made by the psyllid insect *Leuronotata maritima* (Tuthill), a specialist on black mangrove, *Avicennia germinans* (Ilka Feller, in prep.). If an ancestral fergusoninid evolved to preferentially lay eggs in galls formed by nematodes, it might not be much of a jump for the nematode to become phoretic and use the fly for transport to new feeding sites, as is done in many insect‐associated nematodes (Giblin‐Davis, Scheffer, et al. 2004; Borges 2022; van Goor et al. 2023). Oviposition of nematodes along with the fly eggs would give the fly access to galls without having to evolve to create them and the nematodes a way to reach new feeding sites. Reciprocal coevolutionary change between the flies and nematodes would necessarily need to follow for such an interaction to develop into the highly functional and obligate mutualism we see today.

## 5 Conclusion

The intimate and mutualistic interactions between *Fergusonina* and *Fergusobia* and the details of their biology suggest strict transmission of nematodes from female flies to daughters. This is not the case for three densely sampled species that produce galls with multiple female foundresses. Patterns of haplotype discordance within each species indicate intraspecific lateral transfer of nematodes is common. Despite the breakdown in vertical transmission within these species, no patterns of interspecific transfer of nematode haplotypes were observed, and the fly and nematode phylogenies were highly concordant. Although limited, our evidence suggests that the same or related host plant taxa were shared during the few postulated host shifts, suggesting a possible role of plant taxon in facilitating nematode host‐shifting movement between fly lineages.

## Author Contributions

**Sonja J. Scheffer:** conceptualization (lead), data curation (lead), formal analysis (lead), investigation (lead), methodology (lead), project administration (lead), resources (supporting), supervision (lead), validation (lead), visualization (lead), writing – original draft (lead), writing – review and editing (equal). **Matthew L. Lewis:** conceptualization (supporting), data curation (equal), investigation (supporting), methodology (equal), writing – review and editing (equal). **Kerrie A. Davies:** conceptualization (supporting), investigation (supporting), validation (supporting), writing – review and editing (supporting). **Robin M. Giblin‐Davis:** conceptualization (supporting), investigation (supporting), validation (equal), writing – review and editing (equal). **Gary S. Taylor:** conceptualization (supporting), validation (supporting), writing – review and editing (equal). **Leigh A. Nelson:** conceptualization (supporting), investigation (equal), methodology (supporting), writing – review and editing (supporting). **Matthew F. Purcell:** investigation (supporting), validation (supporting), writing – review and editing (supporting). **Jeff R. Makinson:** data curation (supporting), investigation (supporting), writing – review and editing (supporting). **Weimin Ye:** conceptualization (supporting), methodology (supporting), writing – review and editing (supporting). **Kevin E. Omland:** conceptualization (supporting), investigation (supporting), writing – review and editing (supporting). **David K. Yeates:** conceptualization (supporting), funding acquisition (supporting), investigation (supporting), resources (lead), writing – review and editing (supporting).

## Funding

This work was supported by the Commonwealth Scientific and Industrial Research Organisation, McMaster Fellowship program.

## Conflicts of Interest

The authors declare no conflicts of interest.

## Data Availability

All DNA sequences have been deposited into GenBank. Specimen collection information, fly and nematode haplotype designations, and GenBank accession numbers for the haplotype association study are in Appendix Table A2. Collection and GenBank information for specimens in the phylogenetic study are in Appendix Table A3.

## Appendix A

**TABLE A1:** Primers used in this study.

Primer name	Sequence	References
Fly COI		
C1‐J‐2183	CAACATTTATTTTGATTTTTTGG	Simon et al. (1994)
TL2‐N‐3014	TCCATTGCACTAATCTGCCATATTA	Simon et al. (1994)
Nematode COI		
SGNemCOF1	CCTGWTWTGAGTTTTCCKCGT	
COI‐F1a	CCTACTATGATTGGTGGTTTTGGTAATTG	Kanzaki and Futai (2002)
COI‐R2	GTAGCAGCAGTAAAATAAGCACG	Kanzaki and Futai (2002)
Fly and Nematode CAD		
CAD‐54F	GTNGTNTTYCARACNGGNATGGT	Moulton and Wiegmann (2004)
CAD‐405R	GCNGTRTGYTCNGGRTGRAAYTG	Moulton and Wiegmann (2004)
Fly and Nematode PGD		
PGD‐2F	ATHGARTAYGGNGAYATGCA	Regier (2008)
PGD‐2.5R	ATRCAACCNCCRCGCCACAT	Winkler and Mitter (2008)

**TABLE A2:** DNA extraction code, fly species, collection information, gall code, host plant and fly and nematode COI haplotype codes for haplotype associations between *F. daviesae* , *F. taylori* , *F. omlandi*, and their nematodes, see Figures 3, 4, 5, 6.

DNA‐code	FLY	Location	State	Gall	Host (subsp.)	Fly COI hap	Nem COI hap	Fly GenBank	Nem GenBank
FC‐503	*F. omlandi*	Captain's Flat	NSW	07–118	*E. p. (pauciflora)*	foHap.15 n1	woHap.E n1	KY378234	PX056408
FC‐505	*F. omlandi*	Captain's Flat	NSW	07–127	*E. p. (pauciflora)*	foHap.7 n1	woHap.D n2	PX055717	PX056409
FC‐506	*F. omlandi*	Edmonston Rest Area	NSW	07–132	*E. p. (pauciflora)*	foHap.16 n1	woHap.G n12	KY378235	PX056410
FC‐507	*F. omlandi*	Edmonston Rest Area	NSW	07–133	*E. p. (pauciflora)*	foHap.18 n5	woHap.G n12	PX055718	PX056411
FC‐509	*F. omlandi*	Pt. Lookout	NSW	07–148	*E. p. (pauciflora)*	foHap.2 n2	woHap.A n2	KY378237	PX056412
FC‐510	*F. omlandi*	Pt. Lookout	NSW	07–149	*E. p. (pauciflora)*	foHap.5 n1	woHap.C n1	KY378238	PX056413
FC‐512	*F. omlandi*	Pt. Lookout	NSW	07–159	*E. p. (pauciflora)*	foHap.14 n1	woHap.G n12	KY378240	PX056414
FC‐513	*F. omlandi*	Pt. Lookout	NSW	07–165	*E. p. (pauciflora)*	foHap.11 n1	woHap.G n12	PX055719	PX056415
FC‐514	*F. taylori*	Pryor's Hut	ACT	07–355	*E. p. (niphophila)*	ftHap.20 n9	wtHap.E n20	PX055720	PX056416
FC‐521	*F. taylori*	Mt. Buffalo	VIC	07–267	*E. p. (niphophila)*	ftHap.29 n1	—	KY378242	—
FC‐522	*F. taylori*	Mt. Buffalo	VIC	07–279	*E. p. (niphophila)*	ftHap.26 n1	wtHap.C n5	KY378243	PX056417
FC‐524	*F. daviesae*	Falls Creek Village	VIC	07–259	*E. p. (hedraia)*	fdHap.2 n1	wdHap.E n67	PX055721	PX056418
FC‐526	*F. taylori*	Mt. Hotham, w	VIC	07–280	*E. p. (niphophila)*	ftHap.3 n2	—	KY378245	—
FC‐527	*F. daviesae*	Mt. Hotham Village	VIC	07–191	*E. p. (niphophila)*	fdHap.20 n1	wdHap.E n67	PX055722	PX056419
FC‐529	*F. daviesae*	Dinner Plains	VIC	07–201	*E. p. (niphophila)*	fdHap.10 n2	wdHap.E n67	KY378246	PX056420
FC‐530	*F. taylori*	Dinner Plains	VIC	07–286	*E. p. (niphophila)*	ftHap.2 n1	wtHap.C n5	PX055723	PX056421
FC‐531	*F. omlandi*	20 km ne Dinner Plains	VIC	07–197	*E. p. (pauciflora)*	foHap.4 n1	woHap.G n12	PX055724	PX056422
FC‐538	*F. taylori*	Great Lake	TAS	07–316	*E. p. (pauciflora)*	ftHap.13 n1	wtHap.A n8	PX055725	PX056423
FC‐539	*F. taylori*	Great Lake	TAS	07–335	*E. p. (pauciflora)*	fyHap.34 n1	—	KY378248	—
FC‐541	*F. taylori*	Mt. Wellington	TAS	07–318	*E. coccifer*	ftHap.24 n1	wtHap.H n1	KY378250	PX056424
FC‐543	*F. taylori*	Mt. Field	TAS	07–334	*E. coccifer*	ftHap.15 n1	wtHap.A n8	KY378251	PX056425
FC‐550	*F. omlandi*	Captain's Flat	NSW	07–114	*E. p. (pauciflora)*	foHap.18 n5	woHap.G n12	PX055726	PX056427
FC‐551	*F. omlandi*	Captain's Flat	NSW	07–114	*E. p. (pauciflora)*	foHap.18 n5	woHap.G n12	PX055727	PX056428
FC‐553	*F. omlandi*	Captain's Flat	NSW	07–116	*E. p. (pauciflora)*	foHap.18 n5	woHap.G n12	PX055728	PX056429
FC‐555	*F. omlandi*	Captain's Flat	NSW	07–117	*E. p. (pauciflora)*	foHap.6 n1	woHap.G n12	PX055729	PX056430
FC‐556	*F. omlandi*	Captain's Flat	NSW	07–122	*E. p. (pauciflora)*	foHap.18 n5	woHap.G n12	PX055730	PX056431
FC‐557	*F. omlandi*	Captain's Flat	NSW	07–123	*E. p. (pauciflora)*	foHap.13 n1	woHap.D n2	PX055731	PX056432
FC‐558	*F. omlandi*	Captain's Flat	NSW	07–125	*E. p. (pauciflora)*	foHap.2 n2	woHap.G n12	PX055732	PX056433
FC‐559	*F. omlandi*	Captain's Flat	NSW	07–126	*E. p. (pauciflora)*	foHap.12 n1	woHap.H n1	PX055733	PX056434
FC‐561	*F. omlandi*	Pt. Lookout	NSW	07–157	*E. p. (pauciflora)*	foHap.3 n1	woHap.B n4	PX055734	PX056435
FC‐562	*F. omlandi*	Pt. Lookout	NSW	07–158	*E. p. (pauciflora)*	foHap.10 n1	woHap.B n4	PX055735	PX056436
FC‐566	*F. omlandi*	Pt. Lookout	NSW	07–170	*E. p. (pauciflora)*	foHap.1 n1	woHap.A n2	PX055736	PX056437
FC‐567	*F. omlandi*	Pt. Lookout	NSW	07–171	*E. p. (pauciflora)*	foHap.9 n2	woHap.B n4	PX055737	PX056438
FC‐568	*F. omlandi*	Pt. Lookout	NSW	07–172	*E. p. (pauciflora)*	foHap.9 n2	woHap.B n4	PX055738	PX056439
FC‐570	*F. daviesae*	Dead Horse Gap	NSW	07–208	*E. p. (pauciflora)*	fdHap.1 n2	wdHap.A n1	PX055739	PX056440
FC‐572	*F. taylori*	Dead Horse Gap	NSW	07–274	*E. p. (pauciflora)*	ftHap.18 n1	wtHap.E n20	PX055740	PX056441
FC‐574	*F. daviesae*	Falls Creek Village	VIC	07–179	*E. p. (hedraia)*	fdHap.34 n1	wdHap.L n1	PX055741	PX056442
FC‐575	*F. daviesae*	Falls Creek Village	VIC	07–180	*E. p. (hedraia)*	fdHap.22 n31	wdHap.B n1	PX055742	PX056443
FC‐576	*F. daviesae*	Falls Creek Village	VIC	07–181	*E. p. (hedraia)*	fdHap.22 n31	wdHap.G n1	PX055743	PX056444
FC‐578	*F. daviesae*	Falls Creek Village	VIC	07–215	*E. p. (hedraia)*	fdHap.23 n4	wdHap.N n1	PX055744	PX056445
FC‐579	*F. daviesae*	Falls Creek Village	VIC	07–216	*E. p. (hedraia)*	fdHap.23 n4	wdHap.E n67	PX055745	PX056446
FC‐580	*F. daviesae*	Falls Creek Village	VIC	07–217	*E. p. (hedraia)*	fdHap.22 n31	wdHap.C n1	KY378253	KX156141
FC‐581	*F. taylori*	Mt. Hotham, w	VIC	07–277	*E. p. (niphophila)*	ftHap.28 n1	—	PX055746	—
FC‐582	*F. daviesae*	Mt. Hotham Village	VIC	07–192	*E. p. (niphophila)*	fdHap.22 n31	wdHap.E n67	PX055747	PX056447
FC‐584	*F. daviesae*	Mt. Hotham Village	VIC	07–224	*E. p. (niphophila)*	fdHap.32 n1	wdHap.E n67	PX055748	PX056448
FC‐585	*F. taylori*	Mt. Hotham Village	VIC	07–276	*E. p. (niphophila)*	ftHap.17 n1	wtHap.C n5	PX055749	PX056449
FC‐586	*F. taylori*	Mt. Hotham Village	VIC	07–282	*E. p. (niphophila)*	ftHap.5 n1	wtHap.E n20	KY378254	KX156142
FC‐587	*F. taylori*	Dinner Plains	VIC	07–268	*E. p. (niphophila)*	ftHap.6 n2	wtHap.C n5	PX055750	PX056450
FC‐588	*F. taylori*	Mt. Hotham Village	VIC	07–281	*E. p. (niphophila)*	ftHap.6 n2	—	PX055751	—
FC‐589	*F. taylori*	Dinner Plains	VIC	07–270	*E. p. (niphophila)*	ftHap.29 n2	—	PX055752	—
FC‐590	*F. taylori*	Dinner Plains	VIC	07–270	*E. p. (niphophila)*	ftHap.1 n4	—	PX055753	—
FC‐591	*F. taylori*	Dinner Plains	VIC	07–270	*E. p. (niphophila)*	ftHap.29 n2	—	PX055754	—
FC‐592	*F. taylori*	Dinner Plains	VIC	07–275	*E. p. (niphophila)*	ftHap.30 n1	—	PX055755	—
FC‐595	*F. taylori*	Dinner Plains	VIC	07–284	*E. p. (niphophila)*	ftHap.1 n4	wtHap.C n5	PX055756	PX056451
FC‐596	*F. taylori*	Dinner Plains	VIC	07–284	*E. p. (niphophila)*	ftHap.1 n4	—	PX055757	—
FC‐597	*F. taylori*	Dinner Plains	VIC	07–284	*E. p. (niphophila)*	ftHap.1 n4	—	PX055758	—
FC‐602	*F. taylori*	Mt. Wellington	TAS	07–331	*E. coccifer*	ftHap.8 n1	wtHap.B n1	KY378255	PX056452
FC‐604	*F. taylori*	Mt. Wellington	TAS	07–336	*E. coccifer*	ftHap.12 n2	—	PX055759	—
FC‐605	*F. taylori*	Mt. Wellington	TAS	07–337	*E. coccifer*	ftHap.33 n1	—	PX055760	—
FC‐606	*F. taylori*	Mt. Field	TAS	07–329	*E. coccifer*	ftHap.11 n1	wtHap.A n8	KY378257	PX056453
FC‐609	*F. taylori*	Great Lake	TAS	07–319	*E. p. (pauciflora)*	ftHap.12 n2	wtHap.A n8	KY378259	PX056454
FC‐610	*F. taylori*	Great Lake	TAS	07–328	*E. p. (pauciflora)*	ftHap.10 n2	wtHap.A n8	KY378260	PX056455
FC‐611	*F. taylori*	Great Lake	TAS	07–328	*E. p. (pauciflora)*	ftHap.10 n2	—	PX055761	—
FC‐612	*F. taylori*	Great Lake	TAS	07–330	*E. p. (pauciflora)*	ftHap.9 n2	wtHap.A n8	PX055762	PX056456
FC‐613	*F. taylori*	Great Lake	TAS	07–330	*E. p. (pauciflora)*	ftHap.9 n2	wtHap.A n8	PX055763	PX056457
FC‐614	*F. taylori*	Great Lake	TAS	07–332	*E. p. (pauciflora)*	ftHap.14 n1	wtHap.A n8	PX055764	PX056458
FC‐615	*F. taylori*	Great Lake	TAS	07–332	*E. p. (pauciflora)*	ftHap.32 n1	—	PX055765	—
FC‐616	*F. taylori*	Pryor's Hut	ACT	07–355	*E. p. (niphophila)*	ftHap.20 n9	wtHap.E n20	PX055766	PX056459
FC‐617	*F. taylori*	Pryor's Hut	ACT	07–355	*E. p. (niphophila)*	ftHap.20 n9	—	PX055767	—
FC‐618	*F. taylori*	Pryor's Hut	ACT	07–355	*E. p. (niphophila)*	ftHap.20 n9	wtHap.E n20	KY378261	PX056460
FC‐619	*F. taylori*	Pryor's Hut	ACT	07–357	*E. p. (niphophila)*	ftHap.19 n2	—	KY378262	—
FC‐620	*F. taylori*	Pryor's Hut	ACT	07–358	*E. p. (niphophila)*	ftHap.3 n2	wtHap.G n1	PX055768	PX056461
FC‐621	*F. taylori*	Pryor's Hut	ACT	07–360	*E. p. (niphophila)*	ftHap.21 n1	wtHap.E n20	PX055769	PX056462
FC‐622	*F. taylori*	Pryor's Hut	ACT	07–361	*E. p. (niphophila)*	ftHap.19 n2	wtHap.E n20	PX055770	PX056463
FC‐623	*F. taylori*	Pryor's Hut	ACT	07–362	*E. p. (niphophila)*	ftHap.16 n1	wtHap.E n20	PX055771	PX056464
FC‐624	*F. taylori*	Pryor's Hut	ACT	07–363	*E. p. (niphophila)*	ftHap.22 n1	wtHap.D n1	PX055772	PX056465
FC‐625	*F. taylori*	Pryor's Hut	ACT	07–367	*E. p. (niphophila)*	ftHap.7 n1	wtHap.F n1	PX055773	PX056466
FC‐626	*F. taylori*	Pryor's Hut	ACT	07–368	*E. p. (niphophila)*	ftHap.4 n1	wtHap.E n20	PX055774	PX056467
FC‐628	*F. daviesae*	Pryor's Hut	ACT	07–370	*E. p. (niphophila)*	fdHap.24 n1	wdHap.E n67	PX055775	PX056468
FC‐629	*F. taylori*	Mt. Gingera Tract	ACT	07–356	*E. p. (niphophila)*	ftHap.31 n3	—	PX055776	PX056469
FC‐631	*F. daviesae*	Mt. Gingera Tract	ACT	07–376	*E. p. (niphophila)*	fdHap.22 n31	wdHap.K n1	PX055777	PX056470
FC‐632	*F. daviesae*	Mt. Gingera Tract	ACT	07–377	*E. p. (niphophila)*	fdHap.11 n2	wdHap.E n67	KY378263	PX056471
FC‐633	*F. daviesae*	Mt. Gingera Tract	ACT	07–377	*E. p. (niphophila)*	fdHap.11 n2	wdHap.E n67	KY378264	PX056472
FC‐634	*F. daviesae*	Mt. Gingera Tract	ACT	07–378	*E. p. (niphophila)*	fdHap.22 n31	wdHap.E n67	PX055778	PX056473
FC‐636	*F. daviesae*	Mt. Gingera Tract	ACT	07–380	*E. p. (niphophila)*	fdHap.6 n1	wdHap.E n67	PX055779	PX056474
FC‐637	*F. daviesae*	Mt. Gingera Tract	ACT	07–381	*E. p. (niphophila)*	fdHap.22 n31	wdHap.E n67	PX055780	PX056475
FC‐638	*F. daviesae*	Mt. Gingera Tract	ACT	07–382	*E. p. (niphophila)*	fdHap.19 n1	wdHap.I n3	PX055781	PX056476
FC‐639	*F. daviesae*	Mt. Gingera Tract	ACT	07–383	*E. p. (niphophila)*	fdHap.9 n4	wdHap.J n1	PX055782	PX056477
FC‐640	*F. daviesae*	Mt. Gingera Tract	ACT	07–384	*E. p. (niphophila)*	fdHap.22 n31	wdHap.E n67	PX055783	PX056478
FC‐641	*F. daviesae*	Mt. Gingera Tract	ACT	07–385	*E. p. (niphophila)*	fdHap.22 n31	wdHap.E n67	PX055784	PX056479
FC‐642	*F. daviesae*	Mt. Gingera Tract	ACT	07–385	*E. p. (niphophila)*	fdHap.3 n5	wdHap.E n67	PX055785	PX056480
FC‐643	*F. daviesae*	Mt. Gingera Tract	ACT	07–385	*E. p. (niphophila)*	fdHap.3 n5	wdHap.E n67	PX055786	PX056481
FC‐644	*F. daviesae*	Mt. Gingera Tract	ACT	07–385	*E. p. (niphophila)*	fdHap.22 n31	wdHap.E n67	PX055787	PX056482
FC‐645	*F. daviesae*	Mt. Gingera Tract	ACT	07–386	*E. p. (niphophila)*	fdHap.14 n2	wdHap.D n4	PX055788	PX056483
FC‐646	*F. omlandi*	Brindabellas	ACT	07–418	*E. p. (pauciflora)*	foHap.8 n1	woHap.G n12	PX055789	PX056484
FC‐647	*F. omlandi*	Brindabellas	ACT	07–419	*E. p. (pauciflora)*	foHap.17 n1	woHap.F n1	PX055790	PX056485
FC‐649	*F. daviesae*	Perisher	NSW	07–153	*E. p. (niphophila)*	fdHap.30 n1	wdHap.E n67	KY378265	PX056486
FC‐650	*F. daviesae*	Perisher	NSW	07–154	*E. p. (niphophila)*	fdHap.17 n3	wdHap.E n67	PX055791	PX056487
FC‐651	*F. daviesae*	Dead Horse Gap	NSW	07–177	*E. p. (pauciflora)*	fdHap.9 n4	wdHap.E n67	PX055792	PX056488
FC‐652	*F. daviesae*	Dead Horse Gap	NSW	07–204	*E. p. (pauciflora)*	fdHap.33 n1	wdHap.E n67	KY378266	PX056489
FC‐654	*F. daviesae*	Dead Horse Gap	NSW	07–207	*E. p. (pauciflora)*	fdHap.22 n31	wdHap.E n67	PX055793	PX056490
FC‐657	*F. daviesae*	Falls Creek Village	VIC	07–182	*E. p. (hedraia)*	fdHap.14 n2	wdHap.E n67	PX055794	PX056491
FC‐659	*F. daviesae*	Falls Creek Village	VIC	07–184	*E. p. (hedraia)*	fdHap.22 n31	wdHap.E n67	KY378267	PX056492
FC‐660	*F. daviesae*	Falls Creek Village	VIC	07–213	*E. p. (hedraia)*	fdHap.7 n2	wdHap.E n67	PX055795	PX056493
FC‐664	*F. daviesae*	Falls Creek Village	VIC	07–221	*E. p. (hedraia)*	fdHap.22 n31	wdHap.E n67	PX055796	PX056494
FC‐667	*F. daviesae*	Falls Creek Village	VIC	07–261	*E. p. (hedraia)*	fdHap.22 n31	wdHap.E n67	PX055797	PX056495
FC‐668	*F. daviesae*	Falls Creek Village	VIC	07–262	*E. p. (hedraia)*	fdHap.34 n1	wdHap.D n4	PX055798	PX056496
FC‐670	*F. daviesae*	Falls Creek Village	VIC	07–264	*E. p. (hedraia)*	fdHap.18 n3	wdHap.I n3	PX055799	PX056497
FC‐678	*F. daviesae*	Mt. Hotham Village	VIC	07–194	*E. p. (niphophila)*	fdHap.26 n2	wdHap.E n67	PX055800	PX056498
FC‐679	*F. daviesae*	Mt. Hotham Village	VIC	07–222	*E. p. (niphophila)*	fdHap.29 n1	wdHap.D n4	PX055801	PX056499
FC‐680	*F. daviesae*	Mt. Hotham Village	VIC	07–223	*E. p. (niphophila)*	fdHap.22 n31	wdHap.E n67	PX055802	PX056500
FC‐681	*F. daviesae*	Mt. Hotham Village	VIC	07–225	*E. p. (niphophila)*	fdHap.27 n1	wdHap.E n67	PX055803	PX056501
FC‐682	*F. daviesae*	Mt. Hotham Village	VIC	07–227	*E. p. (niphophila)*	fdHap.26 n2	wdHap.E n67	PX055804	PX056502
FC‐683	*F. daviesae*	Mt. Hotham Village	VIC	07–228	*E. p. (niphophila)*	fdHap.5 n3	wdHap.E n67	PX055805	PX056503
FC‐684	*F. daviesae*	Mt. Hotham Village	VIC	07–229	*E. p. (niphophila)*	fdHap.22 n31	wdHap.M n1	PX055806	PX056504
FC‐687	*F. daviesae*	Dinner Plains	VIC	07–199	*E. p. (niphophila)*	fdHap.31 n1	wdHap.E n67	PX055807	PX056505
FC‐688	*F. daviesae*	Dinner Plains	VIC	07–200	*E. p. (niphophila)*	fdHap.23 n4	wdHap.E n67	PX055808	PX056506
FC‐699	*F. daviesae*	Perisher	NSW	07–246	*E. p. (niphophila)*	fdHap.1 n2	wdHap.F n4	PX055809	PX056507
FC‐723	*F. daviesae*	Dead Horse Gap	NSW	07–209	*E. p. (pauciflora)*	fdHap.4 n1	wdHap.E n67	PX055810	PX056508
FC‐790	*F. daviesae*	Mt. Ginini	ACT	08–425	*E. p. (niphophila)*	fdHap.9 n4	wdHap.E n67	PX055811	PX056517
FC‐791	*F. daviesae*	Mt. Ginini	ACT	08–426	*E. p. (niphophila)*	fdHap.23 n4	wdHap.F n4	PX055812	PX056518
FC‐792	*F. daviesae*	Mt. Ginini	ACT	08–427	*E. p. (niphophila)*	fdHap.13 n1	wdHap.E n67	PX055813	PX056519
FC‐793	*F. daviesae*	Mt. Ginini	ACT	08–428	*E. p. (niphophila)*	fdHap.10 n2	wdHap.D n4	PX055814	PX056520
FC‐797	*F. daviesae*	Pryor's Hut	ACT	08–432	*E. p. (niphophila)*	fdHap.9 n4	wdHap.E n67	PX055815	PX056521
FC‐799	*F. daviesae*	Pryor's Hut	ACT	08–434	*E. p. (niphophila)*	fdHap.17 n3	wdHap.E n67	PX055816	PX056522
FC‐800	*F. daviesae*	Pryor's Hut	ACT	08–435	*E. p. (niphophila)*	fdHap.21 n1	wdHap.I n3	PX055817	PX056523
FC‐803	*F. daviesae*	Pryor's Hut	ACT	08–438	*E. p. (niphophila)*	fdHap.8 n2	wdHap.E n67	PX055818	PX056524
FC‐804	*F. daviesae*	Mt. Gingera	ACT	08–439	*E. p. (niphophila)*	fdHap.7 n2	wdHap.E n67	PX055819	PX056525
FC‐805	*F. daviesae*	Mt. Gingera	ACT	08–440	*E. p. (niphophila)*	fdHap.16 n2	wdHap.E n67	PX055820	PX056526
FC‐806	*F. daviesae*	Mt. Gingera	ACT	08–441	*E. p. (niphophila)*	fdHap.16 n2	wdHap.E n67	PX055821	PX056527
FC‐807	*F. daviesae*	Mt. Gingera	ACT	08–442	*E. p. (niphophila)*	fdHap.15 n1	wdHap.F n4	PX055822	PX056528
FC‐808	*F. daviesae*	Mt. Gingera	ACT	08–443	*E. p. (niphophila)*	fdHap.12 n1	wdHap.E n67	PX055823	PX056529
FC‐809	*F. daviesae*	Mt. Gingera	ACT	08–444	*E. p. (niphophila)*	fdHap.22 n31	wdHap.E n67	PX055824	PX056530
FC‐811	*F. daviesae*	Mt. Gingera	ACT	08–446	*E. p. (niphophila)*	fdHap.17 n3	wdHap.E n67	PX055825	PX056531
FC‐812	*F. daviesae*	Mt. Gingera	ACT	08–447	*E. p. (niphophila)*	fdHap.5 n3	wdHap.E n67	PX055826	PX056532
FC‐813	*F. daviesae*	Mt. Gingera	ACT	08–448	*E. p. (niphophila)*	fdHap.5 n3	wdHap.E n67	PX055827	PX056533
FC‐814	*F. daviesae*	Mt. Gingera	ACT	08–449	*E. p. (niphophila)*	fdHap.22 n31	wdHap.H n1	PX055828	PX056534
FC‐815	*F. daviesae*	Mt. Gingera	ACT	08–450	*E. p. (niphophila)*	fdHap.25 n1	wdHap.F n4	PX055829	PX056535
FC‐817	*F. daviesae*	Mt. Gingera	ACT	08–452	*E. p. (niphophila)*	fdHap.18 n3	wdHap.E n67	PX055830	PX056536
FC‐818	*F. daviesae*	Mt. Gingera	ACT	08–453	*E. p. (niphophila)*	fdHap.18 n3	wdHap.E n67	PX055831	PX056537
FC‐820	*F. daviesae*	Mt. Gingera	ACT	08–456	*E. p. (niphophila)*	fdHap.8 n2	wdHap.E n67	PX055832	PX056538
FC‐824	*F. taylori*	Mt. Gingera Tract	ACT	07–356	*E. p. (niphophila)*	ftHap.31 n3	—	PX055833	PX056539
FC‐825	*F. taylori*	Mt. Gingera Tract	ACT	07–356	*E. p. (niphophila)*	ftHap.31 n3	—	PX055834	PX056540
FC‐826	*F. daviesae*	Mt. Gingera Tract	ACT	07–385	*E. p. (niphophila)*	fdHap.22 n31	wdHap.E n67	PX055835	PX056541
FC‐828	*F. daviesae*	Mt. Gingera Tract	ACT	07–385	*E. p. (niphophila)*	fdHap.22 n31	wdHap.E n67	PX055836	PX056542
FC‐829	*F. daviesae*	Mt. Gingera Tract	ACT	07–385	*E. p. (niphophila)*	fdHap.22 n31	wdHap.E n67	PX055837	PX056543
FC‐831	*F. daviesae*	Mt. Gingera Tract	ACT	07–385	*E. p. (niphophila)*	fdHap.22 n31	wdHap.E n67	PX055838	PX056544
FC‐832	*F. daviesae*	Mt. Gingera Tract	ACT	07–385	*E. p. (niphophila)*	fdHap.22 n31	wdHap.E n67	PX055839	PX056545
FC‐833	*F. daviesae*	Mt. Gingera Tract	ACT	07–385	*E. p. (niphophila)*	fdHap.22 n31	wdHap.E n67	PX055840	PX056546
FC‐834	*F. daviesae*	Mt. Gingera Tract	ACT	07–385	*E. p. (niphophila)*	fdHap.3 n5	wdHap.E n67	PX055841	PX056547
FC‐835	*F. daviesae*	Mt. Gingera Tract	ACT	07–385	*E. p. (niphophila)*	fdHap.22 n31	wdHap.E n67	PX055842	PX056548
FC‐837	*F. daviesae*	Mt. Gingera Tract	ACT	07–385	*E. p. (niphophila)*	fdHap.22 n31	wdHap.E n67	PX055843	PX056549
FC‐838	*F. daviesae*	Mt. Gingera Tract	ACT	07–385	*E. p. (niphophila)*	fdHap.22 n31	wdHap.E n67	PX055844	PX056550
FC‐839	*F. daviesae*	Mt. Gingera Tract	ACT	07–385	*E. p. (niphophila)*	fdHap.22 n31	wdHap.E n67	PX055845	PX056551
FC‐840	*F. daviesae*	Mt. Gingera Tract	ACT	07–385	*E. p. (niphophila)*	fdHap.22 n31	wdHap.E n67	PX055846	PX056552
FC‐842	*F. daviesae*	Mt. Gingera Tract	ACT	07–385	*E. p. (niphophila)*	fdHap.22 n31	wdHap.E n67	PX055847	PX056553
FC‐843	*F. daviesae*	Mt. Gingera Tract	ACT	07–385	*E. p. (niphophila)*	fdHap.3 n5	wdHap.E n67	PX055848	PX056554
FC‐844	*F. daviesae*	Mt. Gingera Tract	ACT	07–385	*E. p. (niphophila)*	fdHap.22 n31	wdHap.E n67	PX055849	PX056555
FC‐845	*F. daviesae*	Mt. Gingera Tract	ACT	07–385	*E. p. (niphophila)*	fdHap.3 n5	wdHap.E n67	PX055850	PX056556
FC‐847	*F. taylori*	Pryor's Hut	ACT	07–355	*E. p. (niphophila)*	ftHap.20 n9	—	PX055851	—
FC‐848	*F. taylori*	Pryor's Hut	ACT	07–355	*E. p. (niphophila)*	ftHap.23 n7	wtHap.E n20	PX055852	PX056557
FC‐849	*F. taylori*	Pryor's Hut	ACT	07–355	*E. p. (niphophila)*	ftHap.23 n7	wtHap.E n20	PX055853	PX056558
FC‐850	*F. taylori*	Pryor's Hut	ACT	07–355	*E. p. (niphophila)*	ftHap.20 n9	wtHap.E n20	PX055854	PX056559
FC‐851	*F. taylori*	Pryor's Hut	ACT	07–355	*E. p. (niphophila)*	ftHap.23 n7	wtHap.E n20	PX055855	PX056560
FC‐852	*F. taylori*	Pryor's Hut	ACT	07–355	*E. p. (niphophila)*	ftHap.23 n7	wtHap.E n20	PX055856	PX056561
FC‐853	*F. taylori*	Pryor's Hut	ACT	07–355	*E. p. (niphophila)*	ftHap.23 n7	wtHap.E n20	PX055857	PX056562
FC‐854	*F. taylori*	Pryor's Hut	ACT	07–355	*E. p. (niphophila)*	ftHap.23 n7	wtHap.E n20	PX055858	PX056563
FC‐855	*F. taylori*	Pryor's Hut	ACT	07–355	*E. p. (niphophila)*	ftHap.23 n7	wtHap.E n20	PX055859	PX056564
FC‐856	*F. taylori*	Pryor's Hut	ACT	07–355	*E. p. (niphophila)*	ftHap.20 n9	wtHap.E n20	PX055860	PX056565
FC‐857	*F. taylori*	Pryor's Hut	ACT	07–355	*E. p. (niphophila)*	ftHap.20 n9	wtHap.E n20	PX055861	PX056566
FC‐858	*F. taylori*	Pryor's Hut	ACT	07–355	*E. p. (niphophila)*	ftHap.20 n9	wtHap.E n20	PX055862	PX056567

**TABLE A3:** Collection and GenBank information for specimens used in the large cospeciation phylogenetic analyses (Figures 7 and 8).

Code	Species no.	*Fergusonina* species	Coll. Location	Gall	Host plant	Fly COI	Fly CAD	Fly PGD	Nem COI	Nem CAD	Nem PGD
FC‐122	73	Unknown	Edmund Kennedy Natl. Park, QLD	M‐Shoot Bud	*M. quinquenervia*	KY378206	KY378080	KY378352	PX056403	PX101408	PX068236
FC‐135	62	*F*. n. sp. Taylor 2004	Tea Gardens/Hawks Nest, NSW	U‐Peagall	*M. armillaris*	KY378211	—	KY378353	PX056404	PX101409	PX068237
FC‐136	72	*F*. n. sp. (Davies et al. 2014, 254)	Tea Gardens/Hawks Nest, NSW	U‐Peagall	*M. nodosa*	KY378212	KY378081	KY378354	—	PX101410	PX068238
FC‐137	69	*F*. n. sp. (Davies et al. 2014, 254)	Port Macquarie Racecourse, NSW	U‐Peagall	*M. linariifolia*	KY378213	KY378082	KY378355	PX056405	PX101411	PX068239
FC‐184	61	Unknown	Elizabeth River, so. of Darwin, NT	M‐Shoot Bud	*M. argentea*	KY378228	KY378088	KY378361	PX056406	—	PX068240
FC‐189	35	*F*. n. sp. (Davies, Giblin‐Davis, Ye, Taylor, and Thomas 2013, 168)	Nuriootpa, SA	M‐Flat Leaf	*E. odorata* ?	KY378231	KY378091	KY378362	PX056407	PX101412	—
FC‐508	38	Unknown	Edmonston Rest Area, s of Goulburn, NSW	F‐Flower Bud	*E. pauciflora*	KY378236	KY378092	KY378363	KX156140	PX101413	PX068241
FC‐535	*31*	Unknown	Rd C526, central Tasmania, TAS	M‐Flower Bud	*E. pauciflora*	KY378247	KY378093	KY378364	KX156145	PX101414	PX068242
FC‐544	11	Unknown	Mt. Gingera, ACT	M‐Shoot Bud	*E. stellulata*	KY378252	KY378095	KY378366	PX056426	PX101415	PX068243
FC‐580	*32*	*F. daviesae* Nelson and Yeates	Falls Creek Village, VIC	M‐Shoot Bud	*E. p. (hedraia) or niphophila*	KY378253	KY378096	KY378367	KX156141	PX101416	PX068244
FC‐586	*17*	*F. taylori* Nelson and Yeates	Mt. Hotham Village, VIC	M‐Shoot Bud	*E. p. (niphophila)*	KY378254	—	KY378368	KX156142	PX101417	PX068245
FC‐734	78	*F*. n. sp. (Davies et al. 2014, 231)	Tolga, QLD	Mix	*Syzygium*	KY378281	KY378100	KY378372	PX056509	PX101418	PX068246
FC‐737	33	*F*. n. sp. (Davies, Giblin‐Davis, Ye, Lisnawita Taylor, and Thomas 2013, 133)	Adelaide, SA	M‐Shoot Bud	*E. obliqua*	KY378284	KY378103	KY378375	PX056510	PX101419	PX068247
FC‐746	55	Unknown	So. Goulburn, Federal Highway, NSW	F‐Flower Bud	*Euc*. sp.	KY378292	KY378109	KY378381	PX056511	—	PX068248
FC‐748	54	Unknown	Ben Lomand, NSW	U‐Peagall	*Euc* sp.	KY378293	KY378110	KY378382	PX056512	PX101420	PX068249
FC‐753	26	Unknown	Australian National Univ., ACT	U‐Peagall	*E. mannifera*	KY378297	KY378112	KY378384	PX056513	PX101421	PX068250
FC‐755	22	*F*. n. sp. (Davies and Lloyd 1996, 17)	Canberra, ACT	M‐Flat Leaf	*E. leucoxylon*	KY378299	KY378113	KY378385	PX056514	PX101422	PX068251
FC‐756	56	Unknown	Black Mountain, ACT	M‐Shoot Bud	*E*. sp.	KY378300	KY378114	KY378386	PX056515	PX101423	PX068252
FC‐762	3	*Fergusonina biseta* (Tonnoir, 1937)	NSW	M‐Shoot Bud	*C. maculata*	KY378306	KY378118	KY378390	KX156143	PX101424	PX068253
FC‐764	60	*F. metrosiderosi* Taylor	New Zealand	M‐Shoot Bud	*Metrosideros*	KY378308	KY378119	KY378391	PX056516	PX101425	PX068254
FC‐85	23	Unknown	Meninge, SA	M‐Flat Leaf	*E. leucoxylon*	KY378182	PX068260	—	PX056402	PX101405	PX068233
FC‐91	63	*F. purcelli* Taylor	Daintree Ferry turn, QLD	M‐Shoot Bud	*M. cajuputi*	KY378185	PX068261	KY378345	—	PX101406	PX068234
FC‐95	68	*F. centeri* Taylor	Cairns, QLD	M‐Shoot Bud	*M. leucandendra*	KY378189	KY378072	KY378346	—	PX101407	PX068235
FCV‐123	75	Unknown	Edmond Kennedy Natl. Park, QLD	F‐Flower Bud	*M. quinquenervia*	KY378324	KY378127	KY378395	PX056568	PX101426	PX068255
FCV‐148	11	*F*. n. sp. (Davies et al. 2010, 23)	Mt. Cameron, TAS	M‐Shoot Bud	*E. amygdalina*	KY378326	KY378128	KY378396	PX056569	PX101427	PX068256
FCV‐194	20	*F*. n. sp. (Davies, Giblin‐Davis, Ye, Lisnawita Taylor, and Thomas 2013, 133)	Ku‐Ring‐Gai, NSW	M‐Shoot Bud	*E. haemastoma*	KY378330	KY378129	KY378398	KX156144	PX101428	PX068257
FCV‐201	5	*F*. giblindaviesi Taylor	Saltwater Creek, Cairns, QLD	F‐Flower Bud	*C. ptychocarpa*	KY378335	KY378131	KY378399	PX056570	PX101429	—
FCV‐203	74	*F. turneri* Taylor	Kingscliff, NSW	M‐Shoot Bud	*M. quinquenervia*	KY378336	KY378132	KY378400	PX056571	PX101430	PX068258
FCV‐204	13	*F*. n. sp. (Davies, Giblin‐Davis, Ye, Lisnawita Taylor, and Thomas 2013, 133)	Erindale, SA	M‐Shoot Bud	*E. camaldulensis*	KY378337	KY378133	KY378401	PX056572	PX101431	PX068259
